# Review of the genus *Strumigenys* (Hymenoptera, Formicidae, Myrmicinae) in Hong Kong with the description of three new species and the addition of five native and four introduced species records

**DOI:** 10.3897/zookeys.831.31515

**Published:** 2019-03-18

**Authors:** Kit Lam Tang, Mac P. Pierce, Benoit Guénard

**Affiliations:** 1 School of Biological Sciences, The University of Hong Kong, Kadoorie Biological Sciences, Building, Pok Fu Lam Road, Hong Kong SAR, China The University of Hong Kong Hong Kong China

**Keywords:** Ant diversity, biogeography, China, exotic ants, Hong Kong, *
Strumigenys
*

## Abstract

The species of the ant genus *Strumigenys* Smith, 1860 found in Hong Kong are reviewed based on new sampling efforts performed over the past five years (2014–2018). Prior to this, 12 *Strumigenys* species had been recorded from Hong Kong, all confirmed here. Moreover, we add to this list three newly described species: *S.hirsuta***sp. n.**, *S.lantaui***sp. n.**, and *S.nathistorisoc***sp. n.**, and describe for the first time the worker caste of *S.formosa* Terayama, Lin & Wu, 1995. We report new records for nine additional species, bringing the total number of species to 24, including four newly recorded species (*S.hexamera* Brown, 1958, *S.membranifera* Emery, 1869, *S.nepalensis* Baroni Urbani and De Andrade, 1994, and *S.rogeri* Emery, 1890) which are considered to be introduced to Hong Kong. A global review of the introduced *Strumigenys* species is presented. The taxonomic validity of *S.feae* and *S.formosensis* is discussed in light of new specimen measurements. New ecological information on the swarming periods of 11 species is presented on the basis of year-long sampling of aerial insects. Finally, the importance of our results within Southeast Asia and the need for future sampling efforts in the region is discussed.

## Introduction

With 836 described extant species (Bolton 2018), *Strumigenys* is a hyperdiverse ant genus both taxonomically and morphologically. Though globally distributed, *Strumigenys* is primarily a tropical and subtropical ant genus, notably scarce within more temperate regions (antmaps.org, Janicki et al. 2016; Guénard et al. 2017). The Oriental, Neotropical and Afrotropical realms (sensu Holt et al. 2013) possess the highest species richness globally with 240, 154, and 135 species respectively (Guénard et al. 2017). Within Asia (Oriental, Sino-Japanese, and eastern Palearctic realms), 253 native species and eight introduced species have been recorded. New species discoveries and new species records extending the known distribution range of described species are expected with further sampling (e.g. Eguchi et al. 2011) and exploration of undersampled regions (Guénard et al. 2010; Jaitrong et al. 2016).

*Strumigenys* is typically collected from leaf-litter samples from forests floors, though several species are associated with the accumulated leaf litter in trees (Longino and Nadkarni 1990, Lattke et al. 2018). *Strumigenys* species tend to be associated with primary and secondary forest habitats, but a few species, including several tramp species (e.g. *S.emmae*, *S.membranifera*), are relatively common in open disturbed habitats (Kitahiro et al. 2014). In general, species of *Strumigenys* are specialized predators of Collembola but also often take non-preferred small arthropod prey such as Chilopoda, Diplura, Symphyla, and Acari (Wesson 1936; Wilson 1953; Masuko 1984, 2009a, b), with *Strumigenys* species within a similar community occupying different trophic positions (Mezger and Pfeiffer 2010).

*Strumigenys* is easily distinguishable from other ant genera by the combination of the following characters: small size (TL: ca 2–5 mm), elongate or triangular mandibles, and for many species the presence of spongiform tissues on the propodeal declivity, petiole and postpetiole, and first abdominal segment. In the field, these species can also be identified by their slow-motion, the occasional presence of thanatotic behaviour (Smith 1931; Brown 1949), and small colony sizes ranging from a few dozen to a maximum of 500 workers (Wilson 1959; Terayama et al. 2014). Traditionally, species of *Strumigenys* were included within the tribe Dacetini, but recent molecular phylogenetic work placed them within the tribe Attini as the sister taxon to the phalacromyrmecine ants (Ward et al. 2015).

Despite its small size (1100 km^2^), Hong Kong has a relatively high level of biodiversity, due to its geographic position within continental Asia and subtropical climate. Prior to this study, 11 native and one introduced species of *Strumigenys* had been recorded. Here, we review the species of *Strumigenys* in Hong Kong based on new material collected between 2014 and 2018, describe three new species, and report new records for nine additional species, including four species considered as introduced for the region. We also provide new information on the sociometry of several species collected including reproductive phenology and colony composition.

## Methods and materials

The specimens examined were collected by members of the Insect Biodiversity and Biogeography Laboratory (IBBL) at the University of Hong Kong throughout Hong Kong between 2014 and 2018. A wide range of sampling methods were used, including Winkler extractors, pitfall traps, Malaise traps, and hand collection. Information on altitude, when not recorded in the field with the help of a GPS, was extracted from Google Earth Pro v. 7.1.8.3036 based on the recorded coordinates. Images were taken with a Leica DFC450 camera mounted on a Leica M205 C dissecting microscope. Image montages of the specimens were taken, stacked, enhanced and measured using the Leica Application Suite v. 4.5.

Female alate specimens of *Strumigenys* were collected from two projects using Malaise traps. The first project, led by Mr. Christophe Barthélémy, focuses on wasps and has been run since September 2014 at three main locations (Mai Po Nature Reserve, Pak Sha O, and Ping Chan Shai), with a single Malaise trap set at each location. The majority of traps (75%) are collected every two weeks, with occasional 3- or 4-week periods. While the overall period coverage includes the whole year, traps have been run discontinuously at some sites or during specific years (e.g. interruption from 22 November 2014 to 12 April 2015 in Pak Sha O). The second project focuses on mangrove insect diversity with sampling effort spread across 26 sites and seasonally clustered in two seasons from October to January and May to August. At each site one or two Malaise traps were operating for a period of 2 weeks in both seasons. Due to the peculiar habitat sampled in this study, specimens of *Strumigenys* collected most likely represent transient and not resident species.

**Figure 1.**
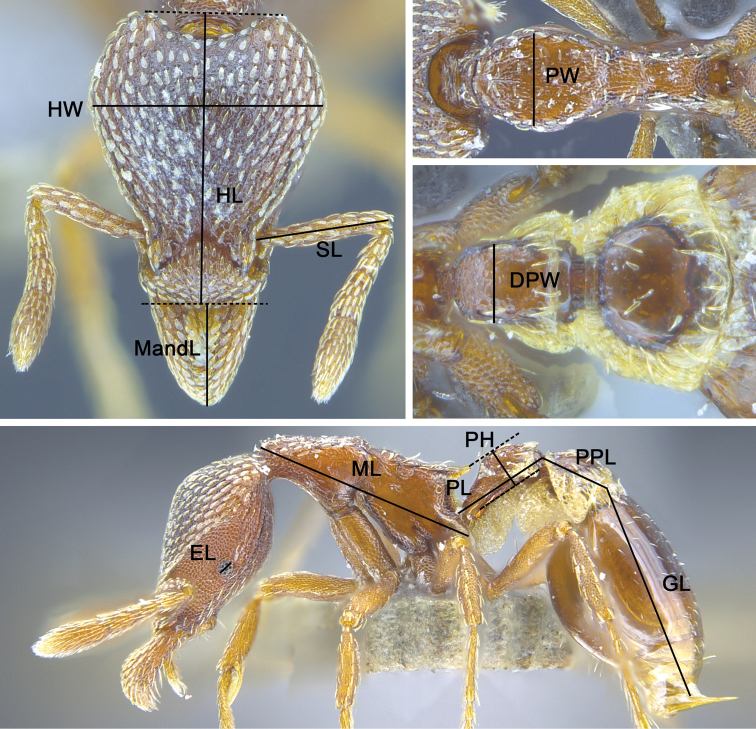
Morphological measurements used. For full definition of each abbreviation, refer to text under the methods and materials section.

Morphological measurements (Fig. 1) and indices are listed below and were used following the standard established for *Strumigenys* (Bolton 2000; Lattke et al. 2018), with the exception of the addition of Postpetiole Length (PPL) and Gaster Length (GL), change to Total Length (TL) with the addition of PPL and GL, and change to Petiolar Height (PH). All measurements are reported in millimetres to the nearest 0.01&nbsp;mm. Sculpture definitions follow Harris (1979).

– Total Length (TL). The total length from the mandibular apex to the posterior margin of abdominal tergite IV. Sum of MandL + HL + ML + PL + PPL + GL.

– Head Length (HL). The length of the head capsule excluding the mandibles, measured in full-face view in a straight line from the midpoint of the anterior clypeal margin to the midpoint of the occipital margin. In species where one or both of these margins is concave, the measurement is taken from the midpoint of a transverse line that spans the apices of the projecting portions.

– Head Width (HW). The maximum width of the head in full-face view, excluding the eyes.

– Mandible Length (MandL). The straight-line length of the mandible at full closure, measured in the same plane for which the HL measurement is taken (i.e. full-face view), from the mandibular apex to the anterior clypeal margin, or to the transverse line connecting the anteriormost points when the margin is concave medially.

– Scape Length (SL). The maximum straight-line length of the scape, excluding the basal constriction or neck that occurs just distal of the condylar bulb. (In species with a hypertrophied subbasal lobe on the scape, SL is measured from the tip of the subbasal lobe to the scape apex.)

– Eye Length (EL). The maximum diameter of the eye.

– Pronotal Width (PW). The maximum width of the pronotum in dorsal view. Projecting tubercles or other cuticular prominences at the pronotal humeral angles, if present, are ignored.

– Mesosoma Length (ML) (= Weber’s Length). The diagonal length of the mesosoma in profile from the point at which the pronotum meets the cervical shield to the posterior basal angle of the metapleuron.

– Petiolar Length (PL). The maximum length of the petiole from the posterior petiolar margin to the point it is obscured by the posteroventral lobes of the propodeum in profile. Spongiform tissues, if present, are ignored.

– Petiolar Height (PH). The maximum distance measured between two parallel lines, one tangent with the node apex and the other tangent with the ventral-most point of the petiole in profile. When the ventral margin is concave upward, then the lower line tangent to the uppermost portion of the curve. Spongiform tissues, if present, are ignored.

– Dorsal Petiolar Width (DPW). The maximum width of the petiolar node in dorsal view.

– Postpetiole Length (PPL). The maximum length of the postpetiole, measured in the same plane for which the PL measurement is taken (i.e. profile view), from the anterior margin to the posterior margin. Spongiform tissues, if present, are ignored.

– Gaster Length (GL). The maximum length of the gaster, measured in the same plane for which the PL measurement is taken (i.e. profile view), from the anterior margin to the posterior margin. Spongiform tissues and sting, if present, are ignored.

– Cephalic Index (CI). HW / HL × 100

– Mandibular Index (MI). MandL / HL × 100

– Scape Index (SI). SL / HW × 100

– Ocular Index (OI). EL / HW × 100

– Lateral Petiolar Index (LPI). PH / PL × 100

– Dorsal Petiolar Index (DPI). DPW / PL × 100

## Results

### Strumigenys canina

Taxon classificationAnimaliaHymenopteraFormicidae

(Brown & Boisvert, 1979) – First recorded in Hong Kong in 1994 (Fellowes 1996)

Pentastruma canina Brown and Boisvert 1979: 203, figs 2–4 (w.q.m.) JAPAN. Palearctic.
Pyramica canina (Brown & Boisvert, 1979). Combination in Pyramica: Bolton 1999: 1673.
Strumigenys canina (Brown & Boisvert, 1979). Combination in Strumigenys: Baroni Urbani and De Andrade 2007: 116.

#### Material examined.

HONG KONG: Central & Western District, LFS Plot 3D, 22.278318N, 114.137804E, 04.01.2016, G. Yong, Winkler, IBBL; Central & Western District, Lung Fu Shan, 22.2783N, 114.138017E, 24.04.2015, R.H. Lee, pitfall trap, IBBL; Central & Western District, Lung Fu Shan, 22.278986N, 114.13717E, 18.11.2014, 211 m, M. Wong, Winkler, 4 Corners, IBBL; Central & Western District, Lung Fu Shan, 22.2790333N, 114.1366202E, 30.12.2015, G. Yong, Winkler, IBBL; Central & Western District, Lung Fu Shan, 22.279201N, 114.137209E, 12.09.2018, B. Guénard, hand collection, IBBL; Central & Western District, Lung Fu Shan, 22.28039N, 114.137830E, 25.11.2014, 295 m, M. Wong, Winkler, 12 Random, IBBL; Central & Western District, The Peak, 22.276038N, 114.141995E, 17.08.2015, R.H. Lee, Winkler, IBBL; Central & Western District, The Peak, 22.2767N, 114.1423E, 17.08.2015, R.H. Lee, Winkler, IBBL; North District, A Ma Wat, 22.5191N, 114.2441E, 19.12.2016, R.H. Lee, Winkler, IBBL; North District, H.W. Hang, 22.52819N, 114.2006E, 14.06.2015, 29 m, T. Tsang, Winkler, IBBL; North District, Lai Chi Wo, 22.527N, 114.258E, 08.05.2015, R.H. Lee, Winkler, IBBL; North District, Sheung Wo Hang, 22.522305N, 114.197237E, 16.06.2015, R.H. Lee, Winkler, IBBL; Sha Tin District, Kam Shan Country Park, 22.3562N, 114.15167E, 18.10.2017, R. Cheung, Winkler, IBBL; Sha Tin District, Kam Shan Country Park, 22.37089N, 114.14839E, 18.10.2017, R. Cheung, Winkler, IBBL; Sha Tin District, Lion Rock, 22.357002N, 114.175047E, 13.07.2015, R.H. Lee, Winkler, IBBL; Sha Tin District, Lion Rock, 22.357002N, 114.175047E, 15.08.2017, R.H. Lee, Winkler, IBBL; Sha Tin District, Lion Rock, 22.35805N, 114.176995E, 13.07.2015, R.H. Lee, Winkler, IBBL; Sha Tin District, Lion Rock, 22.360915N, 114.180028E, 13.07.2015, R.H. Lee, Winkler, IBBL; Sha Tin District, Mau Ping Wood, 22.3844N, 114.241E, 20.10.2015, R.H. Lee, Winkler, IBBL; Sha Tin District, Tai Po Kau Nature Reserve, 22.4281N, 114.1808E, 24.02.2016, B. Guénard, Winkler, IBBL; Sha Tin District, Tai Po Kau Nature Reserve, 22.4285N, 114.1808E, 22.02.2017, B. Guénard, Winkler, IBBL; Sha Tin District, Tai Po Kau, 22.41678N, 114.1878E, 03.07.2015, 317 m, T. Tsang, Winkler, IBBL; Sha Tin District, Tai Po Kau, 22.41841N, 114.1779E, 12.07.2015, 295 m, T. Tsang, Winkler, IBBL; Sha Tin District, Tai Po Kau, 22.422858N, 114.180827E, 14.07.2015, R.H. Lee, Winkler, IBBL; Sha Tin District, Tai Po Kau, 22.42706N, 114.179996E, 14.07.2015, R.H. Lee, Winkler, IBBL; Tai Po District, Kadoorie Centre, 22.4291N, 114.11491E, 08.09.2015, R.H. Lee, Winkler, IBBL; Tai Po District, Kadoorie Centre, 22.4297N, 114.1143E, 08.09.2015, R.H. Lee, Winkler, IBBL; Tai Po District, Kadoorie Farm and Botanic Garden, 22.43076N, 114.1215E, 04.07.2011, 335 m, P. Ward, sifted litter, IBBL; Tai Po District, KFBG, 22.4302N, 114.1192E, 14.09.2015, R.H. Lee, Winkler, IBBL; Tai Po District, Pak Sha O, 22.44743N, 114.3082E, 17.10.2017, R. Cheung / M. Pierce, Winkler, IBBL; Tai Po District, Sha Lo Tong, 22.47708333N, 114.18195E, 28.05.2015, R.H. Lee, Winkler, IBBL; Tai Po District, Sha Lo Tong, 22.4817666N, 114.182833E, 28.05.2015, R.H. Lee, Winkler, IBBL; Tai Po District, Sha Shan, 22.449N, 114.145E, 03.11.2015, R.H. Lee, Winkler, IBBL; Tai Po District, Tai Om, 22.44171N, 114.13518E, 28.02.2018, B. Guénard, Winkler, IBBL; Tai Po District, Tai Om, 22.44184N, 114.134571E, 28.02.2018, B. Guénard, Winkler, IBBL; Tai Po District, Tai Om, 22.4419N, 114.133533E, 05.10.2016, R.H. Lee, Winkler, IBBL; Tai Po District, Tai Om, 22.44214N, 114.13533E, 28.02.2018, B. Guénard, Winkler, IBBL; Tai Po District, Tai To Yan, 22.4538N, 114.11937E, 07.08.2015, R.H. Lee, Winkler, IBBL; Tsuen Wan District, Lin Fa Shan, 22.3956N, 114.0885E, 15.07.2016, R.H. Lee, Winkler, IBBL; 41; Tsuen Wan District, Shing Mun, 22.397083N, 114.1539166E, 14.05.2015, R.H. Lee, Winkler, IBBL; Tsuen Wan District, Shing Mun, 22.39755N, 114.15385E, 14.05.2015, R.H. Lee, Winkler, IBBL; Tsuen Wan District, Shing Mun, 22.39845N, 114.1628E, 24.08.2015, 367 m, T. Tsang, Winkler, IBBL; Tuen Mun District, Castle Peak, 22.390117N, 113.955767E, 30.06.2015, R.H. Lee, pitfall trap, IBBL; Yuen Long District, Ng Tung Chai, 22.429589N, 114.131276405085E, 01.11.2016, R.H. Lee, pitfall trap, IBBL; Yuen Long District, Ng Tung Chai, 22.429589N, 114.131276405085E, 01.11.2016, R.H. Lee, Winkler, IBBL.

#### Ecology.

**Figure 2.**
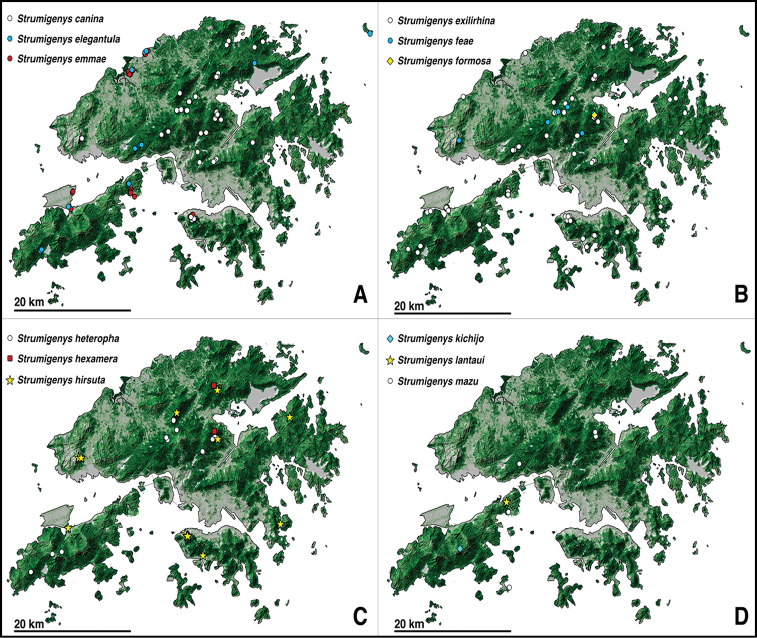
Distribution of *Strumigenys* species in Hong Kong **A***S.canina*, *S.elegantula*, and *S.emmae***B&nbsp**;*S.&nbsp;exilirhina*, *S.feae*, and *S.formosa***C***S.heteropha*, *S.hexamera*, and *S.hirsuta* sp. n. **D***S.kichijo*, *S.lantaui* sp. n., and *S.mazu*. Circles represent species previously recorded from Hong Kong, diamonds represent newly recorded species, and stars represent new species. Newly recorded introduced species are shown with red squares, and previously recorded introduced species are shown with red circles. Green shaded portions of the map correspond with higher levels of tree cover, and grey with lower levels of tree cover.

One of the most common species of *Strumigenys* in Hong Kong (Fig. 2), collected in a variety of habitats including trees along roadsides, shrubland, tree plantation (*Lophostemonconfertus* Wilson & Waterh.), bamboo forest, secondary forest and Feng Shui woods. Known elevation range in Hong Kong from 29 to 474 m. In a Winkler sample collected on October 8^th^, 2018, 149 workers, 3 queens, 1 larva of a gyne, 24 alate gynes, and 1 male were collected altogether, potentially belonging to the same colony.

### Strumigenys elegantula

Taxon classificationAnimaliaHymenopteraFormicidae

(Terayama & Kubota, 1989) – First recorded in Hong Kong in 1993 (Fellowes 1996)

Smithistruma elegantula Terayama and Kubota 1989: 788, figs 23–27 (w.q.) TAIWAN. Indomalaya.
Pyramica elegantula (Terayama & Kubota, 1989). Combination in Pyramica: Bolton 1999: 1673.
Strumigenys elegantula (Terayama & Kubota, 1989). Combination in Strumigenys: Baroni Urbani and De Andrade 2007: 119.

#### Material examined.

HONG KONG: Islands District, Shek Pik, 22.2309N, 113.8861E, 18.08.2015, R.H. Lee, Winkler, IBBL; Islands District, Shek Pik, 22.233N, 113.888E, 18.08.2015, R.H. Lee, pitfall trap, IBBL; North District, Lai Chi Wo, 22.527N, 114.258E, 08.05.2015, R.H. Lee, Winkler, IBBL; Tai Po District, Sha Lo Tong, 22.47708N, 114.18195E, 28.05.2015, R.H. Lee, pitfall trap, IBBL; Tai Po District, Tung Ping Chau, 22.5382N, 114.4365E, 02.10.2017, R. Cheung / B. Morgan, Winkler, IBBL; Tai Po District, Wu Kau Tang, 22.49645N, 114.2441E, 25.10.2015, 29 m, T. Tsang, Winkler, IBBL; Tsuen Wan District, Tai Lam Country Park, 22.38091N, 114.05324E, 08.11.2017, R. Cheung / M. Pierce, Winkler, IBBL; Tsuen Wan District, Tai Lam Country Park, 22.38109N, 114.05511E, 08.11.2017, R. Cheung / M. Pierce, Winkler, IBBL; Yuen Long District, Lok Ma Chau, 22.51192N, 114.06064E, 29.05.2018, 1 m, M. Wong, pitfall trap, IBBL; Yuen Long District, Lok Ma Chau, 22.51378N, 114.06301E, 29.05.2018, 1 m, M. Wong, pitfall trap, IBBL.

#### Ecology.

While this species is seldom collected in Hong Kong (Fig. 2), it was found across a wide range of areas and habitats including managed grasslands, trees along roadsides, shrubland, secondary forest, and Feng Shui woods. Known elevation range in Hong Kong is from 1 to 254 m.

### Strumigenys emmae

Taxon classificationAnimaliaHymenopteraFormicidae

(Emery, 1890) – First recorded in Hong Kong in 1993 (Fellowes 1999)

Epitritus emmae Emery 1890: 70, pl. 8, fig. 6 (w.) ANTILLES. Neotropic.
Quadristruma emmae (Emery, 1890). Combination in Quadristruma: Brown 1949: 48.
Strumigenys emmae (Emery, 1890). Combination in Strumigenys: Bolton 1999: 1674.

#### Material examined.

HONG KONG: Central & Western District, HKU campus, near Chemistry building, 22.28275N, 114.13981E, 29.05.2015, C. Wang, Winkler, IBBL; Central & Western District, HKU CYT, 22.28245N, 114.14042E, 07.01.2016, G. Yong, Winkler, IBBL; Central & Western District, The Peak, 22.27604N, 114.14199E, 17.08.2015, R.H. Lee, Winkler, IBBL; Islands District, Disneyland, 22.30812N, 114.04318E, 27.07.2016, B. Guénard, Gut content Water Dragon, IBBL; Yuen Long District, Lok Ma Chau, 22.50942N, 114.06076E, 19.06.2018, 0 m, M. Wong, pitfall trap, IBBL; Yuen Long District, Lok Ma Chau, 22.50963N, 114.06053E, 19.06.2018, 1 m, M. Wong, pitfall trap, IBBL; Yuen Long District, Mai Po, 22.48023N, 114.03576E, 30.07.2018, 10 m, M. Wong, pitfall trap, IBBL; Yuen Long District, Mai Po, 22.48048N, 114.03514E, 30.07.2018, 10 m, M. Wong, pitfall trap, IBBL; Yuen Long District, Mai Po, 22.48153N, 114.03289E, 30.07.2018, 10 m, M. Wong, pitfall trap, IBBL; Yuen Long District, Mai Po, 22.48482N, 114.03335E, 07.08.2018, 1 m, M. Wong, pitfall trap, IBBL; Yuen Long District, Mai Po, 22.4858N, 114.0391E, 26.10.2016, R.H. Lee, pitfall trap, IBBL; Yuen Long District, Mai Po, 22.4868N, 114.0409E, 26.10.2016, R.H. Lee, pitfall trap, IBBL.

#### Ecology.

An introduced species, likely originating from the Australian realm (Bolton 2000), found mostly in disturbed habitats, including managed grasslands, isolated patches of urban trees, and with a single record from a secondary forest but located slightly over 100 m from urban habitations. Common in the Mai Po Nature Reserve (Fig. 2), a heavily disturbed landscape managed for bird populations. Known elevation range in Hong Kong is from 1 to 407 m. A single alate gyne was collected between June 27 and July 11 in a Malaise trap located within a mangrove area.

### Strumigenys exilirhina

Taxon classificationAnimaliaHymenopteraFormicidae

Bolton, 2000 – First recorded in Hong Kong in 2000 (Bolton 2000)

Strumigenys exilirhina Bolton 2000: 881 (w.q.) NEPAL. Indomalaya.

#### Material examined.

HONG KONG: Central & Western District, HKU Campus, 22.28216N, 114.13829E, 19.11.2014, 113 m, M. Wong, Winkler, 12 Random, IBBL; Central & Western District, HKU campus, near Robert Black, 22.282129N, 114.138105E, 01.05.2015, C. Wang, Winkler, IBBL; Central & Western District, HKU CYT, 22.2824528N, 114.140429E, 07.01.2016, G. Yong, Winkler, IBBL; Central & Western District, LFS Plot 1 B–C, 22.277134N, 114.134792E, 28.12.2015, G. Yong, Winkler, IBBL; Central & Western District, LFS Plot 1 C, 22.277134N, 114.134806E, 28.12.2015, G. Yong, Winkler, IBBL; Central & Western District, Lung Fu Shan Park, N, E, 03.05.2015, C. Wang, Winkler, IBBL; Central & Western District, Lung Fu Shan, 22.276729N, 114.136693E, 24.11.2014, 295 m, M. Wong, Winkler, 12 Random, IBBL; Central & Western District, Lung Fu Shan, 22.28039N, 114.137830E, 25.11.2014, 156 m, M. Wong, Winkler, 4 Corners, IBBL; Central & Western District, Lung Fu Shan, 22.28221N, 114.133476E, 13.11.2014, 115 m, M. Wong, Winkler, 12 Random, IBBL; Central & Western District, Plot 1 B–C, 22.27713N, 114.13479E, 08.01.2016, G. Yong, Winkler, IBBL; Central & Western District, The Peak, 22.276038N, 114.141995E, 17.08.2015, R.H. Lee, Winkler, IBBL; Eastern District, Tai Tam, 22.259933N, 114.22009E, 27.07.2015, R.H. Lee, Winkler, IBBL; Islands District, Lamma Island, 22.20363N, 114.13599E, 14.09.2017, R.H. Lee, Winkler, IBBL; Islands District, Luk Tei Tong, 22.26233N, 113.99066E, 25.10.2016, R.H. Lee, pitfall trap, IBBL; Islands District, Pak Ngan Heung, 22.27099N, 113.98911E, 25.10.2016, R.H. Lee, pitfall trap, IBBL; Islands District, Shek Pik, 22.230898N, 113.88606E, 18.08.2015, R.H. Lee, Winkler, IBBL; Islands District, Shek Pik, 22.240075N, 113.89041E, 18.08.2015, R.H. Lee, Winkler, IBBL; North District, A Ma Wat, 22.5191N, 114.2441E, 19.12.2016, R.H. Lee, Winkler, IBBL; North District, Kuk Po Sam To, 22.523977N, 114.2355E, 15.11.2016, R.H. Lee, Winkler, IBBL; North District, Kuk Po San Uk, 22.529123N, 114.234675E, 15.11.2016, R.H. Lee, Winkler, IBBL; North District, Sheung Wo Hang, 22.52203N, 114.1962E, 12.06.2015, 71 m, T. Tsang, Winkler, IBBL; North District, Sheung Wo Hang, 22.52232N, 114.1972E, 16.06.2015, 99 m, T. Tsang, Winkler, IBBL; Sai Kung District, Pak Tam Chung, 22.400962N, 114.327163E, 05.06.2015, R.H. Lee, Winkler, IBBL; Sha Tin District, Lion Rock, 22.35805N, 114.176995E, 13.07.2015, R.H. Lee, Winkler, IBBL; Sha Tin District, Lion Rock, 22.360915N, 114.180028E, 13.07.2015, R.H. Lee, Winkler, IBBL; Sha Tin District, Lion Rock, 22.36121N, 114.181997E, 13.07.2015, R.H. Lee, Winkler, IBBL; Sha Tin District, Mui Tsz Lam wood, 22.389185N, 114.234462E, 04.10.2016, R.H. Lee, pitfall trap, IBBL; Sha Tin District, Tai Po Kau Nature Reserve, 22.4285N, 114.1808E, 22.02.2017, B. Guénard, Winkler, IBBL; Sha Tin District, Tai Po Kau, 22.41678N, 114.1878E, 03.07.2015, 317 m, T. Tsang, Winkler, IBBL; Sha Tin District, Tai Po Kau, 22.422858N, 114.180827E, 14.07.2015, R.H. Lee, Winkler, IBBL; Sha Tin District, Tai Po Kau, 22.42402N, 114.18029E, 14.07.2015, R.H. Lee, Winkler, IBBL; Sha Tin District, Tai Po Kau, 22.426138N, 114.181783E, 14.07.2015, R.H. Lee, Winkler, IBBL; Sha Tin District, Tai Po Kau, 22.427285N, 114.181298E, 16.09.2015, B. Guénard, hand collection, IBBL; Southern District, Lam Long Shan, 22.23887N, 114.16864E, 20.09.2017, R.H. Lee, Winkler, IBBL; Southern District, Nam Fung Road, 22.2546N, 114.1833E, 20.08.2016, R.H. Lee, pitfall trap, IBBL; Southern District, Nam Fung Road, 22.25519N, 114.1818E, 28.09.2015, 120 m, T. Tsang, Winkler, IBBL; Southern District, Nam Fung Road, 22.25554N, 114.1802E, 01.10.2015, 110 m, T. Tsang, Winkler, IBBL; Tai Po District, Kadoorie Farm and Botanic Garden, 22.43076N, 114.1215E, 04.07.2011, 335 m, P. Ward, sifted litter, IBBL; Tai Po District, KFBG, 22.4302N, 114.1192E, 14.09.2015, R.H. Lee, Winkler, IBBL; Tai Po District, Sha Lo Tong, 22.477083N, 114.18195E, 28.05.2015, R.H. Lee, Winkler, IBBL; Tai Po District, Sha Lo Tong, 22.477N, 114.1797E, 28.05.2015, R.H. Lee, Winkler, IBBL; Tai Po District, Sha Lo Tong, 22.481767N, 114.18283E, 28.05.2015, R.H. Lee, Winkler, IBBL; Tai Po District, Sha Shan, 22.449N, 114.145E, 03.11.2015, R.H. Lee, Winkler, IBBL; Tai Po District, Tai Om, 22.4419N, 114.133533E, 05.10.2016, R.H. Lee, Winkler, IBBL; Tai Po District, To Kwa Peng, 22.42901N, 114.3336E, 25.05.2018, 1 m, R. Cheung / C. Taylor, Malaise trap, IBBL; Tsuen Wan District, Ha Lin Fa Shan, 22.39664N, 114.1019E, 31.07.2015, 355 m, T. Tsang, Winkler, IBBL; Tsuen Wan District, Shing Mun, 22.396783N, 114.1531E, 14.05.2015, R.H. Lee, pitfall trap, IBBL; Tsuen Wan District, Shing Mun, 22.39678N, 114.1531E, 23.08.2015, 238 m, T. Tsang, Winkler, IBBL; Tsuen Wan District, Shing Mun, 22.39693N, 114.153E, 17.05.2016, R.H. Lee, Winkler, IBBL; Tsuen Wan District, Shing Mun, 22.397083N, 114.1539166E, 14.05.2015, R.H. Lee, Winkler, IBBL; Yuen Long District, Kap Lung, 22.41596N, 114.1038E, 11.09.2015, 288 m, T. Tsang, Winkler, IBBL; Yuen Long District, Ng Tung Chai, 22.42959N, 114.13128E, 01.11.2016, R.H. Lee, Winkler, IBBL; Yuen Long District, Sheung Pak Nai, 22.45151N, 113.96213E, 28.05.2018, 1 m, R. Cheung / C. Taylor, Malaise trap, IBBL; Yuen Long District, Sheung Tin Liu Ha, 22.44348N, 114.114E, 03.08.2015, 106 m, T. Tsang, Winkler, IBBL.

#### Ecology.

This is one of the most common species of *Strumigenys* in Hong Kong (Fig. 2). It has been collected in a variety of habitats including disturbed urban forests, tree plantations (*Lophostemonconfertus* Wilson & Waterh.), shrubland, secondary forest, and Feng Shui woods. The known elevation range in Hong Kong for this species is from 1 to 407 m.

### Strumigenys feae

Taxon classificationAnimaliaHymenopteraFormicidae

Emery, 1895 – First recorded in Hong Kong as S. formosensis in Bolton (2000); see comments below

Fig. 3A–B

Strumigenys feae Emery 1895: 473 (w.q.) MYANMAR. Indomalaya.

#### Material examined.

HONG KONG: Tai Po District, Tai Om, 22.43681N, 114.1373E, 07.08.2015, 138 m, T. Tsang, Winkler, IBBL; Tsuen Wan District, Shing Mun, 22.40027N, 114.161E, 04.09.2015, 366 m, T. Tsang, Winkler, IBBL; Tuen Mun District, Castle Peak, 22.389935N, 113.954937E, 30.06.2015, R.H. Lee, pitfall trap, IBBL; Yuen Long District, Kap Lung, 22.41596N, 114.1038E, 11.09.2015, 288 m, T. Tsang, Winkler, IBBL; Sha Tin District, Tai Po Kau Nature Reserve, 22.4285N, 114.1808E, 22.02.2017, B. Guénard, Winkler, IBBL; Tai Po District, Kadoorie Farm and Botanic Garden, 22.43076N, 114.1215E, 04.07.2011, 335 m, P. Ward, sifted litter, IBBL.

#### Measurements.

Workers (*n* = 2): TL 3.1–3.3, HL 0.81–0.87, HW 0.52–0.55, MandL 0.39–0.41, SL 0.52–0.53, EL 0.061–0.062, PW 0.26–0.28, ML 0.82, PL 0.30, PH 0.15–0.16, DPW 0.11–0.12, PPL 0.19–0.20, GL 0.58–0.71, CI 63–64, MI 47–48, SI 96–100, OI 11–12, LPI 51–52, DPI 37–40.

#### Ecology.

In Hong Kong, *S.feae* was collected within tree plantations of *Lophostemonconfertus* Wilson & Waterh. and in secondary forests, with elevation ranging from 138 to 457 m.

#### Comments.

While *S.formosensis* (Forel, 1912) has been recorded from Hong Kong (Bolton 2000), we consider these records as *S.feae*. *Strumigenysformosensis* was initially described as a subspecies of *S.feae*, and Brown (1949: 24) raised *S.formosensis* to the species level without strong justification and without examining specimens of *S.feae*, writing: “Although I have seen no specimens of Emery’s Burmese species *feae*, I am arbitrarily raising the Taiwan form to species rank.”, on the basis of Forel’s description of *S.formosensis* having small propodeal teeth and a strongly concave posterior mesosomal dorsum, with this latter information absent in Emery’s description of *S.feae*. The examination of the pictures of the type specimen of *S.feae* available on AntWeb (CASENT0904951), however, show the presence of a concavity between the mesonotum and propodeum, and with propodeal spines of the type of *S.formosensis* (CASENT0909309) indistinctly smaller than *S.feae*.

The revised descriptions of *S.feae* and *S.formosensis* by Bolton (2000) also revealed no clear distinction between them except the difference in morphological measurements, the length and morphology of the preapical teeth (“not directed medially but instead so strongly inclined toward the apicodorsal tooth that its proximal margin forms a single continuous line with the inner mandibular margin” for *S.formosensis*), and brief mentioning of the maximum diameter of the eye compared to the width of the scape (“slightly greater” for *S.feae* and “equal to or slightly less” for *S.formosensis*), with the rest of the descriptions almost identical to one another.

**Figure 3.**
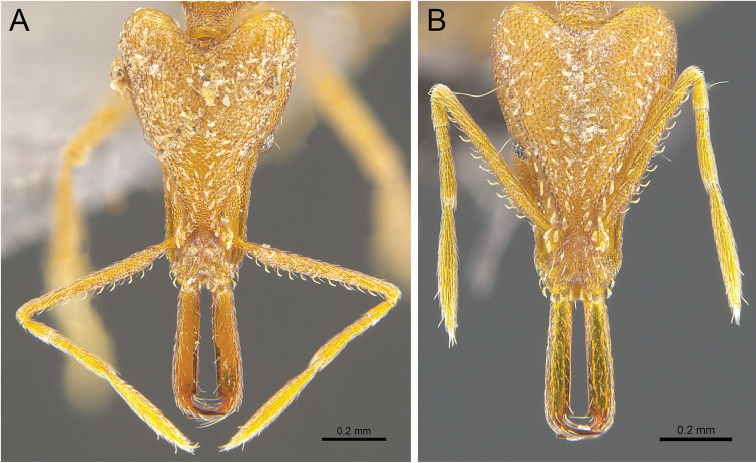
*Strumigenysfeae/formosensis***A** worker (ANTWEB1017082) full-face view **B** worker (RHL01266) full-face view.

Specimens collected in Hong Kong could not be assigned to either *S.feae* or *S.formosensis* without ambiguity under the current descriptions. Preapical teeth are neither fully directed medially as in *S.feae*, nor with a single continuous proximal margin as in *S.formosensis* (Fig. 3). Morphological measurements also give little additional information. Measurements of the specimen ANTWEB1017082 (Fig. 3A), which has more forward-inclined preapical teeth, fall within the norm of *S.formosensis* as expected, specimen RHL01266 (Fig. 3B) with more medially-directed preapical teeth has some of its measurements closer to *S.formosensis* than to *S.feae* (Table 1). Considering the fact that *S.formosensis* was raised to its current species level somewhat arbitrarily, the validity of *S.formosensis* as a species would require further investigation using specimens from a wider geographic range than is available for this study.

**Table 1.** Morphological measurements of *S.feae* and *S.formosensis* comparing information on specimens presented in Bolton (2000) and specimens collected in Hong Kong. For additional information, refer to text under *Strumigenysfeae*.

Species/Specimens	HL	HW	SL	MandL	PW	ML	CI	SI	MI
**Measurements (in mm) from Bolton (2000)**									
* S. feae *	0.75–0.80	0.47–0.52	0.48–0.50	0.33–0.36	0.27–0.28	0.72–0.80	61–68	94–102	41–46
* S. formosensis *	0.84–0.87	0.54–0.56	0.52–0.54	0.39–0.40	0.25–0.28	0.75–0.78	63–65	93–98	46–47
**Measurements (in mm) from two Hong Kong specimens**									
ANTWEB1017082	0.87	0.55	0.53	0.41	0.28	0.82	63	96	47
RHL01266	0.81	0.52	0.52	0.39	0.26	0.82	64	100	48

### Strumigenys formosa

Taxon classificationAnimaliaHymenopteraFormicidae

(Terayama, Lin & Wu, 1995) - new record

Fig. 4A–C

Epitritus formosus Terayama et al., 1995: 85, figs 1–4 (q.) TAIWAN. Indomalaya.
Pyramica formosus (Terayama et al., 1995). Combination in Pyramica: Bolton 1999: 1672.
Strumigenys formosa (Terayama et al., 1995). Combination in Strumigenys: Baroni Urbani and De Andrade 2007: 120.

#### Material examined.

HONG KONG, Sha Tin District, Tai Po Kau Nature Reserve, 22.426138N 114.181783E, 162 m, 6.VII.2017, R.H. Lee, RHL03476, pitfall trap, IBBL.

#### Measurements.

Worker (*n* = 1): TL 1.6, HL 0.35, HW 0.38, MandL 0.13, SL 0.18, EL 0.024, PW 0.24, ML 0.41, PL 0.18, PH 0.10, DPW 0.12, PPL 0.11, GL 0.41, CI 109, MI 37, SI 47, OI 6, LPI 57, DPI 68.

This species has been described from two queens collected in 1988 in Nantou County, Taiwan. To the best of our knowledge, no additional records have been reported in the following 30 years. A single worker was collected in Hong Kong which fits the morphological characteristics and size of *S.formosa*. In the absence of nest series, assigning this worker to this species might be uncertain, however, the extreme rarity of this species in Hong Kong and Taiwan limits the likelihood of collecting nest series. As a result, in the presence of several convergent characters, we assign the worker collected to *S.formosa*. Complete description and diagnosis are provided below.

#### Worker description.

**Figure 4.**
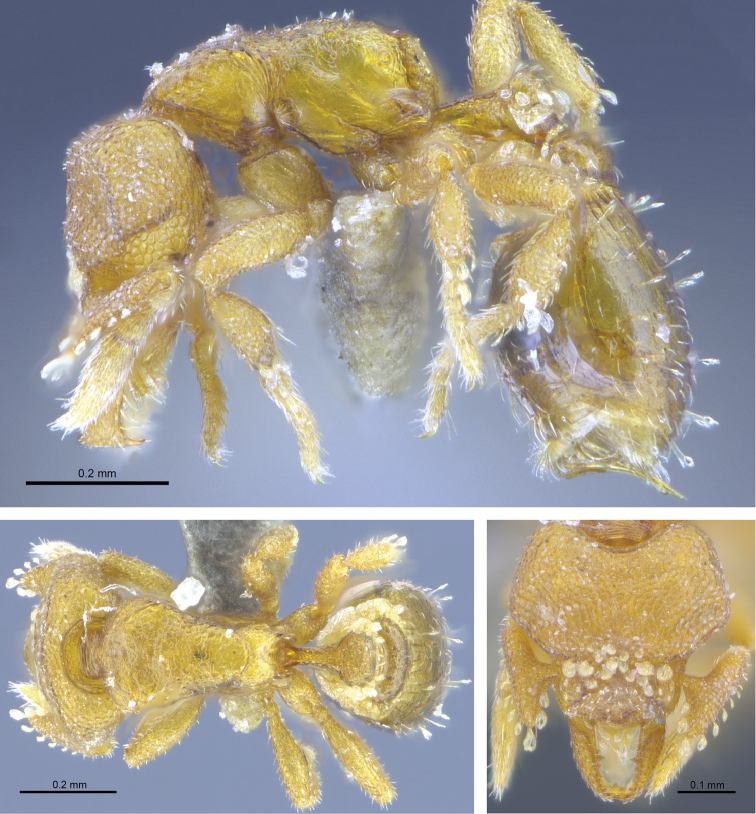
*Strumigenysformosa***A–C** worker (RHL003476) **A** profile view **B** dorsal view **C** full-face view.

(Fig. 4) ***Head*.** In full-face view, head slightly longer than wide with its widest portion near its mid-length. Occipital margin deeply, evenly concave; occipital corners well developed and flattened on their apical portion, then forming a rounded angle with lateral margins. Posterolateral margins divergent on more than half of their length, then more abruptly converging towards the centre of head. Anteromedian clypeal margin slightly convex. Scapes with a well-developed subbasal lobe on their anterior portion. Mandibles elongate and curvilinear. Inner margin of mandibles without spoon-shaped hairs and with teeth clearly visible. In the mid-part of each mandible, a single denticle present, followed by a well-developed tooth and further three denticles all similar to the first. In profile view, apical portion of mandibles distinctly enlarged and with apicoventral tooth distinct (but not in full-face view) and longer than other teeth. In anterior view of the mandibles, enlarged extremity of mandibles composed of a single apicodorsal tooth followed by a series of very fine, compact, baleen-like mandibular setae. Eyes present but reduced and indistinct, on lateroventral position.

***Mesosoma.*** In profile view, dorsum of mesosoma continuous and slightly concave on its mesonotum portion. Pronotomesopleural suture visible and extending on about one-third on the height of the pronotum. Fine lamellae of spongiform tissues present on propodeal declivity, with its upper posterior portion slightly acute as in female holotype. Metapleural gland bulla well developed. In dorsal view, thorax trapezoidal with pronotum much wider than mesonotum and propodeum. Anterior margin of pronotum convex and forming rounded angles with lateral margins.

***Waist segments.*** Petiolar peduncle long, its lateral margins slightly concave in shape when seen in dorsal view. In profile view, petiolar node low and rounded. In profile view, postpetiole lower than petiole. In dorsal view, postpetiole distinctly larger than petiole, bean shaped, and fully surrounded by spongiform tissues.

***Pilosity.*** On posterior half of head, pilosity limited to a few short J-shaped hairs present on lateral margin of head and oriented apically when head observed in full-face view. More short J-shaped hairs visible in profile view, slightly denser on particular on the posterior margin of head. In full head view, as for the female reproductive caste, frontal lobes covered by about 15 appressed large orbicular hairs arranged longitudinally. Clypeus with sparse presence of small to medium-sized spoon-shaped hairs. Anterior clypeal margin with four spoon-shaped hairs pointing forward and directed towards the mid-point of the clypeus, with central hairs significantly larger than those present on lateral margins. Spoon-shaped hairs completely lacking on mandibles but with finer pubescence present. On scapes, spoon-shaped hairs present on lateral margins and arranged in a crescendo fashion from smaller hairs present from about two-third of the scape on its apical to larger hairs present on the subbasal lobes; all pointing anterodorsally towards the apex of subbasal antennal lobe. In profile view, a few short, acute and erected hairs visible on mesonotum and anterodorsal part of the propodeum. Legs with numerous suberected fine and long hairs present on femurs, with apical portion of femurs bearing a few spoon-shaped hairs. Petiolar node with a continuous collar of four large spoon-shaped hairs oriented backwards and extending from the lateral margin of the petiole at about its midpoint to its dorsal portion, with their size increasing posteriorly. Other thick hairs present on dorsal portion of the petiole and oriented backwards. Sparse erected spoon-shaped hairs present on gaster significantly longer with their basal portion elongated and thin. Fine elongated simple hairs present on sternites and arranged transversely.

***Sculpture.*** Head finely aerolated in all visible portions, including scapes, but not on mandibles. Aerolate sculptures particularly well defined around the eyes, when specimen observed in profile view. In profile view, pronotum mostly reticulated at the exception of its most ventral region which is smooth. Mesopleuron and metapleuron almost entirely smooth and shiny. Posterodorsal part of the propodeum reticulated. In dorsal view, thorax with coarse reticulated sculpture. Coxa, femur and tibia aerolated. In profile view, petiole mainly reticulated at the exception of the anterior part of the petiolar peduncle smooth. In dorsal view, petiole clearly reticulated. Visible part of postpetiolar node smooth and shiny. Gaster entirely smooth and shiny, with only short longitudinal striae present on anterior portion of the dorsal part of the tergite of the fourth metasomal segment.

***Colouration.*** Bright yellow for most of the body at the exception of the gaster which is slightly darker.

#### Comments.

The specimen clearly shares all characters of the *S.murphyi*-group (Bolton 2000) for which five species occur within the Oriental realm. The absence of flattened hairs on the inner margin of the mandibles distinguishes it from *S.dyschima*, *S.hemisobek*, and *S.murphyi*. All hairs on the scape are curved toward the basis of the scape, in contrast to *S.nannosobek*, which has hairs that are pointing towards the apex. On the Hong Kong specimen, the posterior margin of the head is deeply concave, contrary to other species of the *S.murphyi*-group except for *S.formosa* (based on the queen description), which also lacks flattened hairs on the inner margin of the mandibles and has a similar disposition and orientation of spoon-shaped hairs on the scape.

#### Geographic range.

Hong Kong, Taiwan. This record of *S.formosa* represents the second record for this species and the first outside of Taiwan. Therefore, this species should not be considered as endemic to Taiwan.

#### Ecology.

The only worker known from Hong Kong (Fig. 2) was collected in a secondary forest at an elevation of 162 m.

### Strumigenys heteropha

Taxon classificationAnimaliaHymenopteraFormicidae

Bolton, 2000 – First recorded in Hong Kong in 1996 (Bolton 2000)

Strumigenys heteropha Bolton 2000: 758 (w.) CHINA. Palearctic.

#### Material examined.

HONG KONG: Islands District, Tei Tong Tsai, 22.25707N, 113.92628E, 29.11.2016, R.H. Lee, Winkler, IBBL; Sha Tin District, Tai Po Kau, 22.41698N, 114.1789E, 03.07.2015, 337 m, T. Tsang, Winkler, IBBL; Sha Tin District, Tai Po Kau, 22.42007N, 114.1829E, 02.07.2015, 291 m, T. Tsang, Winkler, IBBL; Tai Po District, KFBG, 22.4302N, 114.1192E, 14.09.2015, R.H. Lee, Winkler, IBBL; Tsuen Wan District, Shing Mun, 22.39845N, 114.1628E, 24.08.2015, 367 m, T. Tsang, Winkler, IBBL; Tsuen Wan District, Shing Mun, 22.39962N, 114.162E, 12.08.2015, 355 m, T. Tsang, Winkler, IBBL; Yuen Long District, Kap Lung, 22.41596N, 114.1038E, 11.09.2015, 288 m, T. Tsang, Winkler, IBBL; Yuen Long District, Kap Lung, 22.41931N, 114.1018E, 24.09.2015, 190 m, T. Tsang, Winkler, IBBL; Yuen Long District, Sheung Tin Liu Ha, 22.44348N, 114.114E, 03.08.2015, 106 m, T. Tsang, Winkler, IBBL.

#### Ecology.

This species was collected in several closed-canopy habitats including tree plantations of *Lophostemonconfertus* Wilson & Waterh., secondary forests and Feng Shui woods (Fig. 2) with an elevation range from 106 to 367 m.

### Strumigenys hexamera

Taxon classificationAnimaliaHymenopteraFormicidae

(Brown, 1958) - new record

Fig. 5A–C

Epitritus hexamerus Brown 1958: 70, figs 1–3 (w.q.) JAPAN. Palearctic.
Pyramica hexamerus (Brown, 1958). Combination in Pyramica: Bolton 1999: 1672.
Strumigenys hexamera (Brown, 1958). Combination in Strumigenys: Baroni Urbani and De Andrade 2007: 122.

#### Material examined.

HONG KONG: Sha Tin District, Tai Po Kau, 22.42841N, 114.18197E, 22.02.2017, 160 m, R.H. Lee, Winkler, IBBL; Tai Po District, Ping Shan Chai, 22.486N, 114.187E, 25.03.2017, 142 m, C. Barthélémy, Malaise trap, IBBL.

#### Measurements.

Worker (*n* = 1): HL 0.47, HW 0.50, MandL 0.18, SL 0.22, EL 0.036, PW 0.27, ML 0.53, PL 0.23, PH 0.12, DPW 0.15, PPL 0.16, CI 106, MI 38, SI 44, OI 7, LPI 51, DPI 63. Queen (*n* = 1): TL 2.7, HL 0.54, HW 0.60, MandL 0.20, SL 0.25, EL 0.10, PW 0.37, ML 0.71, PL 0.34, PH 0.17, DPW 0.22, PPL 0.18, GL 0.78, CI 111, MI 37, SI 42, OI 17, LPI 48, DPI 65.

#### Geographic range.

***Native***: Japan (mainland and Ryukyu Islands), South Korea, Taiwan.

***Introduced***: Hong Kong, Ogasawara Islands (Japan), United States.

#### Ecology.

This is a rare species in Hong Kong with only two records, both from secondary forests at elevations of 142 and 160 m (Fig. 2). This species seems to have small monogynous colonies of about 35 individuals (Terayama et al. 2014).

#### Comments.

The record of *S.hexamera* in Hong Kong represents the first record of this species for continental China. This species is known as an introduced species in Southeast USA (Alabama, Florida, Louisiana, and Mississippi), and was reported as introduced within the Ogasawara Islands (Shindo 1979). Here we tentatively classify this species as introduced to Hong Kong in light of its tramp characteristics, including its known thelytokous reproductive strategy (Masuko 2013), and the lack of previous collections in Hong Kong or other parts of mainland China. However, for this species, as for many tramp species across Asia, further study is needed to determine their exact origin and the extent of their native range.

**Figure 5.**
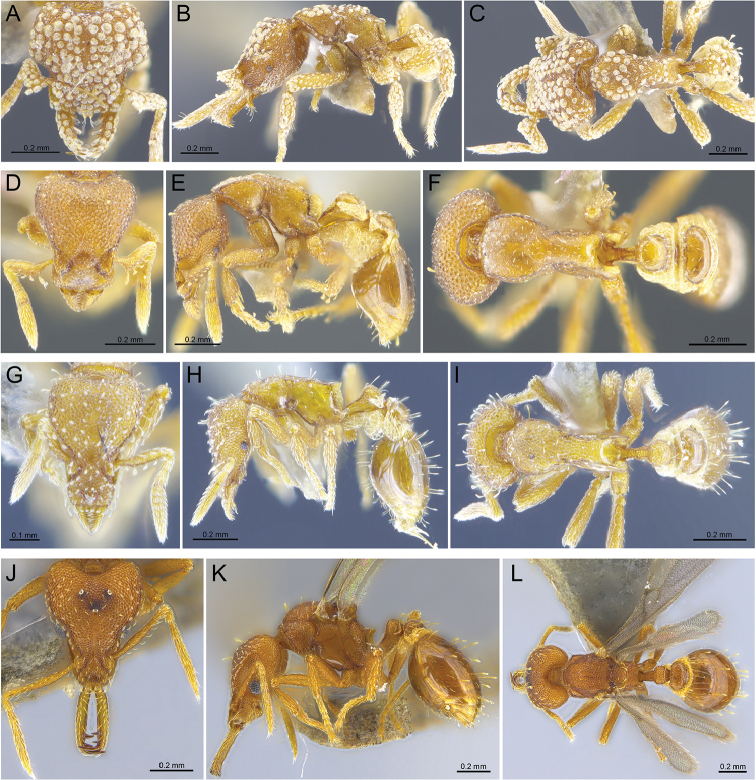
New introduced species records for Hong Kong, in full-face, profile, and dorsal view, respectively **A–C** worker of *S.hexamera* (RHL003477) **D–F** worker of *S.membranifera* (BMW02021) **G–I&nbsp**;worker of *S.nepalensis* (BMW02392) **J–L** queen of *S.rogeri* (ANTWEB1013909).

### Strumigenys hirsuta sp. n.

Taxon classificationAnimaliaHymenopteraFormicidae

http://zoobank.org/CEDE445A-A4B3-4368-AA45-EBEFE44A7483

Figs 6A–C , 7A–C

#### Diagnosis.

Dorsolateral margin of head in full-face view with at most 4 freely laterally projecting flagellate hairs: 1 on the upper scrobe margin posterior to the level of eye, 1 at apicoscrobal position, 0–2 posterior to this on the lateral margin of occipital lobe. Cephalic dorsum, dorsal mesosoma and side of pronotum densely and strongly reticulate-punctate; metapleuron and side of propodeum reticulate-punctate but weaker and fainter than on the dorsum; katepisternum mostly smooth and shining. Dorsal and ventral surfaces of femur with numerous fine erect to suberect hairs. SI 61–63.

#### Type material.

Holotype worker: Hong Kong, Hong Kong Island, Lung Fu Shan, 22.27899N, 114.13717E, 211 m, 18 November 2014 (M. Wong) (collection code F2W-m^2^) [IBBL, ANTWEB1009855]. Paratype workers (*n* = 5): same data as holotype worker. Holotype queen: same data as holotype worker.

#### Non-type material examined.

1 queen and 17 workers. HONG KONG: Central & Western District, Lung Fu Shan, 22.279201N, 114.137209E, 12.09.2018, B. Guénard, hand collection, IBBL; Central & Western District, Lung Fu Shan, 22.28039N, 114.13783E, 25.11.2014, 156 m, M. Wong, Winkler, 12 Random, IBBL; Central & Western District, Lung Fu Shan, 22.28221N, 114.133476E, 13.11.2014, 115 m, M. Wong, Winkler, IBBL; Central & Western District, Lung Fu Shan, R.H. Lee, pitfall trap, IBBL; Islands District, Tung Chung, 22.2907N, 113.9371E, 1 m, B.M. Worthington, Winkler, 12 Random, IBBL; Islands District, Tung Chung, 22.2907N, 113.9371E, B.M. Worthington, Winkler, 4 Corners, IBBL; Sai Kung District, Clear Water Bay Country Park, 22.29618N, 114.29239E, 24.10.2017, 113 m, R. Cheung / M. Pierce, Winkler, 12 Random, IBBL; Sai Kung District, Pak Sha O, 22.44743N, 114.3082E, 17.11.2017, 135 m, R. Cheung / M. Pierce, Winkler, 4 Corners, IBBL; Sha Tin District, Tai Po Kau, 22.41678N, 114.1878E, 03.07.2015, 317 m, T. Tsang, Winkler, IBBL; Tai Po District, Ping Shan Chai, 22.486N, 114.187E, 04.06.2016, C. Barthélémy, Malaise trap, IBBL; Tai Po District, Tai To Yan, 22.4538N, 114.11937E, 07.08.2015, 459 m, R.H. Lee, Winkler, IBBL; Tuen Mun District, Castle Peak, 22.389935N, 113.954937E, 30.06.2015, 457 m, R.H. Lee, pitfall trap, IBBL; Tuen Mun District, Castle Peak, 22.39012N, 113.958983E, 30.06.2015, 204 m, R.H. Lee, Winkler, IBBL

#### Measurements.

Holotype worker: TL 3.1, HL 0.74, HW 0.55, MandL 0.30, SL 0.35, EL 0.054, PW 0.31, ML 0.79, PL 0.35, PH 0.15, DPW 0.14, PPL 0.22, GL 0.69, CI 75, MI 41, SI 63, OI 10, LPI 42, DPI 40. Paratype workers (*n* = 5): TL 2.9–3.1, HL 0.71–0.74, HW 0.53–0.55, MandL 0.29–0.30, SL 0.34–0.35, EL 0.050–0.057, PW 0.29–0.31, ML 0.74–0.78, PL 0.32–0.34, PH 0.13–0.15, DPW 0.14, PPL 0.20–0.21, GL 0.63–0.70, CI 73–75, MI 40–42, SI 63–65, OI 9–11, LPI 41–44, DPI 43–45. Holotype queen: TL 3.6, HL 0.78, HW 0.59, MandL 0.31, SL 0.37, EL 0.12, PW 0.38, ML 0.89, PL 0.44, PH 0.19, DPW 0.19, PPL 0.23, GL 0.90, CI 76, MI 40, SI 62, OI 21, LPI 43, DPI 44.

#### Worker description.

**Figure 6.**
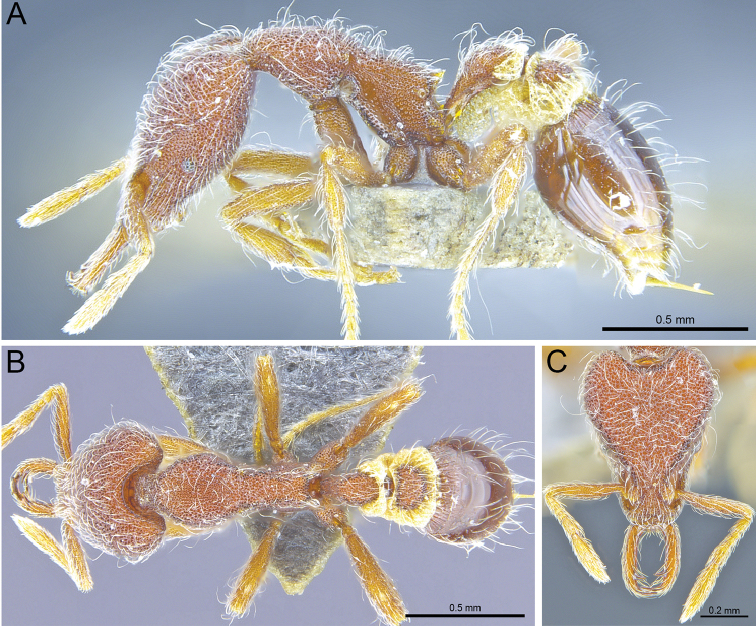
*Strumigenyshirsuta* sp. n. **A–C** Holotype worker (ANTWEB1009855) **A** Profile view **B** dorsal view **C** full-face view.

(Fig. 6A–C). ***Head.*** In full-face view occipital margin evenly concave; occipital corners well developed and bluntly rounded; anterior clypeal margin transverse to very shallowly concave across its width. Scapes subcylindrical, marginated but not converging anteriorly to form a thin lamella at the leading edge. Mandible in full-face view long, narrow and elongated, with an elongated preapical tooth located close to the apicodorsal tooth; at the basal third of their length diverging from one another and curving inward, then running straight and parallel at the middle third, and curving inward and converging at the apical third; width of mandible fairly constant from the basal portion to where the preapical tooth first arose; the preapical tooth about the same to slightly longer than the width of mandible at the point where the tooth arises; apicodorsal tooth is markedly longer than apicoventral tooth.

***Mesosoma.*** In profile pronotal dorsum broadly convex, with the rest of the dorsum of the mesosoma more or less flat transversely; pronotum marginate dorsolaterally. In dorsal view, lateral margins of the pronotum evenly convex. Propodeal teeth short, triangular and acute, and not subtended by lamella.

***Waist segments.*** Petiole in profile claviform, the node long and low; the peduncle grade evenly into the node without a marked change of slope; node in dorsal view longer than broad. Disc of the postpetiole in dorsal view very slightly broader than long, and slightly shorter than petiolar node. Spongiform tissues present on both petiole and postpetiole; ventral lobes of petiole and postpetiole extensive. In profile view, spongiform tissues present ventrally on the peduncle of the petiole notably larger than that under the petiolar node portion, and markedly thicker than the height of the peduncle. Lateral lobe of petiole restricted to posterior half of the node in profile; in dorsal view present along the posterior margin of the petiolar node and surrounding the disc of postpetiole.

***Pilosity.*** Dorsolateral margin of head in full-face view with at most 4 pairs of freely laterally projecting flagellate hairs: 1 on the upper scrobe margin posterior to the level of eye, 1 at apicoscrobal position, 0–2 posterior to this on the lateral margin of occipital lobe. Cephalic dorsum, against ground pilosity of short, suberect to decumbent, simple hairs, with several erect flagellate hairs close to the occipital margin but without erect hairs anterior to this. Leading edge of scape with apically directed, decumbent simple hairs. Pronotal humeral hair long, flagellate or looped apically. Dorsum and side of mesosoma covered with ground pilosity of short, suberect hairs arising and curving in various directions. Dorsal and ventral surfaces of femur with numerous fine erect to suberect hairs against ground pilosity of appressed hairs; dorsal surface of tibia and basitarsus with 1–4 long filiform erect hairs on each segment. Petiolar node and postpetiole with numerous erect to suberect, flagellate hairs against ground pilosity of posteriorly directed, shorter decumbent hairs; first gastral tergite with numerous curved to subflagellate erect hairs.

***Sculpture.*** Cephalic dorsum densely and strongly reticulate-punctate. Dorsal mesosoma and side of pronotum densely and strongly reticulate-punctate, occasionally with very weak and faint rugulose; metapleuron and side of propodeum also reticulate-punctate to punctate, with reticulation limited to the dorsal half of the propodeum, and weaker and fainter than on the dorsum or side of pronotum, sometimes even partially smooth and shining; katepisternum mostly smooth and shining, with some light punctation and vestiges of sculpture around the margins. Anterior coxae with weak transverse rugulae. Petiole and disc of postpetiole densely and strongly reticulate-punctate. Basigastral costulae arise across the entire width of tergite, short and limited to the basal third or fourth of tergite.

#### Gyne description.

**Figure 7.**
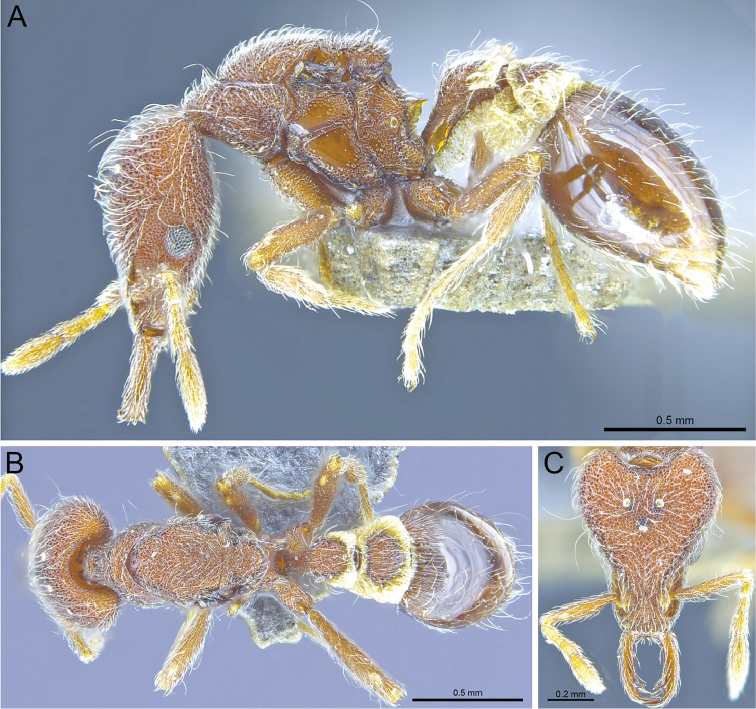
*Strumigenyshirsuta* sp. n. **A–C** holotype queen (ANTWEB1009854) **A** profile view **B** dorsal view **C** full-face view.

(Fig. 7A–C) Similar to all points to the worker caste except for the reproductive caste morphological characters (presence of 3 ocelli, enlarged eyes and thorax), and the following: in profile view, most of anepisternum and katepisternum distinctly smooth and shiny.

#### Comments.

*Strumigenyshirsuta* is a member of the *caniophanes*-complex in the *S.caniophanes*-group and shares all the characters (Bolton 2000). There are six other species (*S.dipsas*, *S.paraposta*, *S.lacunosa*, *S.benulia*, *S.daithma*, and *S.pliocera*) from the species group that also have unsculptured (or at least not completely sculptured) katepisternum as in *S.hirsuta*. In comparison, *S.dipsas* and *S.paraposta* both have reticulate-rugulose sculpture with fine punctulate on the cephalic dorsum, and predominant longitudinal rugulose sculpture on the pronotal dorsum (instead of simply predominant reticulate-punctate on both cephalic and pronotal dorsum as in *S.hirsuta*); *S.lacunosa*, *S.benulia*, and *S.daithma* have their entire pleurae and side of propodeum mostly smooth (instead of katepisternum only); *S.pliocera* has no erect hairs on the dorsal surface of its hind femur, unlike *S.hirsuta*.

*Strumigenyshirsuta* (HL 0.71–0.74, HW 0.53–0.55, ML 0.74–0.78) is a smaller species than *S.dipsas* (HL 0.80–0.86, ML 0.87–0.90) and *S.pliocera* (HL 0.89, ML 0.90); and a larger species than *S.benulia* (HL 0.56, HW 0.39, ML 0.56). *Strumigenyshirsuta* (SI 63–65) also has a markedly relatively shorter scape than those 6 species from the *caniophanes*-complex: *S.benulia* (SI 72), *S.dipsas* (SI 73–76), *S.paraposta* (SI 73–78), *S.lacunosa* (SI 75), *S.daithma* (SI 85), and *S.pliocera* (SI 84).

#### Etymology.

The species is named for the multiple standing and convoluted hairs present on most of the body.

#### Geographic range.

Hong Kong.

#### Ecology.

*Strumigenyshirsuta* appears to be widespread in Hong Kong and has been collected from multiple locations in Hong Kong Island, the New Territories, and Lantau Island (Fig. 2). Considering the widespread distribution of this species in Hong Kong and its association with disturbed secondary forests or forest remnants, we hypothesize that the geographic range of this species might extend further. It seems likely that this species also occurs in Guangdong province (China). The known elevation range is from 1 to 459 m.

### Strumigenys kichijo

Taxon classificationAnimaliaHymenopteraFormicidae

(Terayama, Lin & Wu, 1996) - new record

Fig. 8A–C

Smithistruma kichijo Terayama Lin and Wu 1996: 335, figs 23–25, 28, 29 (w.) TAIWAN. Indomalaya.
Pyramica kichijo (Terayama, Lin & Wu, 1996). Combination in Pyramica: Bolton 1999: 1673.
Strumigenys kichijo (Terayama, Lin & Wu, 1996). Combination in Strumigenys: Baroni Urbani and De Andrade 2007: 122.

#### Material examined.

HONG KONG: Islands District, Sunset Peak, 22.26112N, 113.956332E, 572 m, 28.03.2016, R.H. Lee, Winkler, IBBL.

#### Measurement.

Workers (*n* = 3): TL 2.5–2.6, HL 0.59, HW 0.46–0.47, MandL 0.14–0.16, SL 0.32–0.33, EL 0.045–0.049, PW 0.30, ML 0.61–0.66, PL 0.31–0.33, PH 0.14–0.15, DPW 0.17–0.18, PPL 0.23–0.24, GL 0.60–0.62, CI 78–80, MI 24–27, SI 70, OI 10, LPI 45–48, DPI 53–58.

#### Geographic range.

Bhutan, China (Fujian, Hunan, Yunnan, Hong Kong), Japan, Taiwan, Thailand, Vietnam.

#### Ecology.

This is a rare species for Hong Kong with a single worker collected (Fig.&nbsp;2) in secondary forest at a relatively high elevation (572 m).

#### Comments.

This is a widespread species in Asia, though rarely collected. The new record from Hong Kong fits within the known range of this species, which ranges in Asia from Hunan (north) to Thailand (south), and from Bhutan (west) to Okinawa (east).

**Figure 8.**
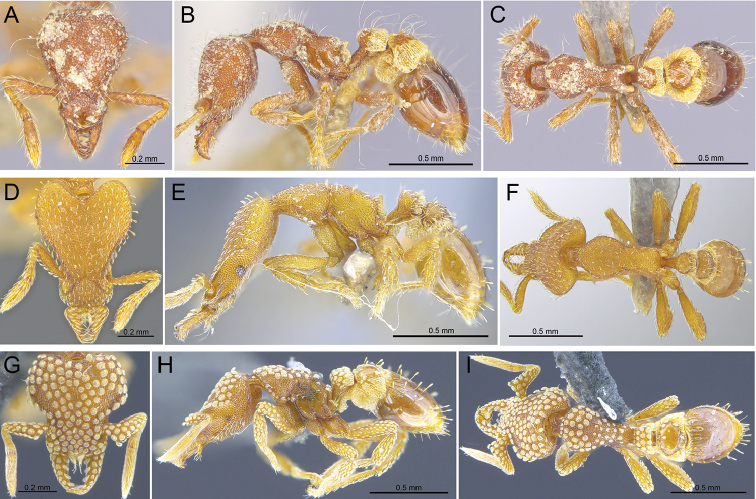
New species records for Hong Kong, in full-face, profile, and dorsal view, respectively **A–C** worker of *S.kichijo* (RHL003471) **D–F** worker of *S.sydorata* (RHL003404) **G–I** worker of *S.tisiphone* (RHL02818).

### Strumigenys lantaui sp. n.

Taxon classificationAnimaliaHymenopteraFormicidae

http://zoobank.org/A577ADFF-6CCB-4E1B-A7FE-6F803A0C64EC

Fig. 9A–D

#### Diagnosis.

Anterior clypeal margin medially shallowly convex with 6 anteriorly projecting strap-like hairs; lateral margin each with 3 anteriorly directed spatulate hairs that are smaller than those on the clypeal margin. Mandibles without preapical tooth. Antenna 4-segmented. Orbicular hairs present on dorsal surface of scapes, head, pronotum and mesonotum. Mesonotum in profile not forming a differentiated surface between pronotum and propodeum.

#### Type material.

Holotype worker: Hong Kong, Lantau Island, Penny’s Bay, 22.3271N, 114.0335E, 9 m, 25 October 2017 (M. Pierce) (collection code WC-PB-NON-06), Winkler [IBBL, ANTWEB1009620]. Paratype workers (*n* = 2): same data as holotype.

#### Measurements.

Holotype worker: TL 1.6, HL 0.36, HW 0.31, MandL 0.10, SL 0.18, EL 0.026, PW 0.20, ML 0.39, PL 0.19, PH 0.09, DPW 0.11, PPL 0.13, GL 0.40, CI 86, MI 28, SI 58, OI 8, LPI 51, DPI 57. Paratype workers (*n* = 2): TL 1.6–1.7, HL 0.37, HW 0.32–0.33, MandL 0.10–0.11, SL 0.19, EL 0.019–0.022, PW 0.21, ML 0.41, PL 0.19, PH 0.10, DPW 0.11, PPL 0.13–0.14, GL 0.43–0.45, CI 86–89, MI 27–30, SI 58–59, OI 6–7, LPI 53, DPI 55–57.

#### Worker description.

***Head.*** In full-face view occipital margin broadly concave; occipital corner evenly rounded; lateral margin broadly convex and slightly diverging from one another, then forming blunt angle with the strongly converging upper scrobe margin; anterior clypeal margin broadly concave across its width. In profile vertex at or near its highest point evenly curved and convex, without a raised transverse crest. Antenna 4-segmented; scape dorsoventrally flattened and board; subbasal angle expanded anteriorly into a large subbasal lobe. Mandible in full-face view narrow, elongate, curvilinear and without preapical tooth. Proximal to apices with a prominent diastema between the mandibles, through which the labral lobes are visible. Apex of mandible with a nearly vertical series of minute teeth or denticles; apicoventral teeth markedly enlarged.

**Figure 9.**
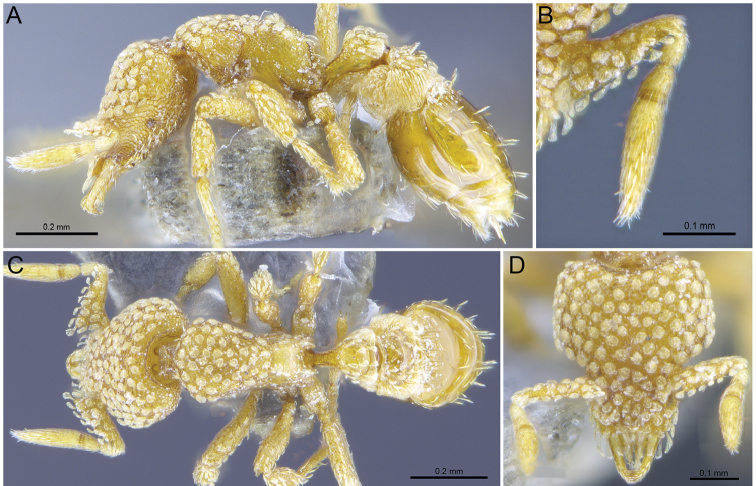
*Strumigenyslantaui* sp. n. **A–D** holotype worker (ANTWEB1009620) **A** profile view **B** close-up on the antenna **C** dorsal view **D** full-face view.

***Mesosoma.*** In profile view the dorsum of mesosoma broadly, shallowly convex; pronotum bluntly marginate laterally; mesonotum not forming a differentiated surface between pronotum and propodeum; in dorsal view lateral margin with a bluntly rounded angle on each side and meet anteriorly into a broadly convex anterior margin. Propodeum unarmed, declivity with a lamella running down each side.

***Waist segments.*** Petiole in profile elongate and subclavate; peduncle does not grade evenly into the node; node in dorsal view long about the same as broad. Disc of postpetiole in dorsal view broader than long and slightly shorter or about the same as petiolar node. Spongiform tissues present on both petiole and postpetiole; ventral lobes of postpetiole in particular extensive; lateral lobe of petiole restricted to posterior portion of the node in profile; in dorsal view present along the posterior margin of the petiolar node, and surrounding disc of postpetiole.

***Pilosity.*** Anterior clypeal margin with 6 anteriorly projecting strap-like hairs; lateral margin each with 3 anteriorly directed spatulate hairs that are smaller than those on clypeal margin. Leading edge of scape with row of basally directed spatulate hairs. Orbicular hairs present on dorsal surface of scapes and all over the cephalic dorsum, including clypeal dorsum, and promesonotal dorsum; dorsal surface of mandibles with short appressed simple or spatulate hairs. Dorsal surfaces of tibiae with short appressed spatulate hairs. Petiolar node and disc of postpetiole with posteriorly directed spatulate hairs, and at most a few decumbent to suberect short simple hairs on the disc of postpetiole; first gastral tergite with numerous short, simple standing hairs.

***Sculpture.*** Cephalic dorsum, including surface of antennal scrobe and antenna, areolate. Dorsum of mesosoma and side of pronotum areolate to densely reticulate-punctate, and with fine reticulopunctate; mesopleuron and side of propodeum mostly smooth and shining, while in some specimens those can appear opaquer but with undefined sculpture. Dorsum of petiolar node and disc of postpetiole generally smooth and shining; basigastral costulae short and arise across entire width of tergite.

#### Comments.

*Strumigenyslantaui* is a member of the *argiola*-complex in the *S.argiola*-group and shares all the characters (Bolton 2000). *S.lantaui* is well distinguished from all other Oriental species in the species group by its 4-segmented antennae, and absence of any scale-like, orbicular, or spoon-shaped hairs on the surface of the mandibles. In addition, unlike *S.hirashimai* or *S.lachesis*, projecting strap-like hairs on the anterior clypeal margin are not directed nor curved towards the midline. It is unlike *S.hexamera* and *S.tisiphone*, in the absence of preapical teeth, and the mesonotum, in profile, not forming a differentiated surface between pronotum and propodeum.

*Strumigenyslantaui* (HL 0.36–0.37, HW 0.31–0.33, ML 0.39–0.41) is also a smaller species than all other Oriental species in the species group: *S.hirashimai* (HL 0.40–0.46, HW 0.36–0.40, ML 0.42–0.48), *S.lachesis* (HL 0.39, HW 0.39, ML 0.42), *S.hexamera* (HL 0.50–0.53, HW 0.53–0.55, ML 0.57–0.60), *S.tisiphone* (HL 0.50, HW 0.48, ML 0.53), and *S.sinensis* (HL 0.52, HW 0.46, ML 0.50).

#### Etymology.

This species is named after Lantau Island, the type locality and currently only known location for the species.

#### Geographic range.

Hong Kong.

#### Ecology.

*Strumigenyslantaui* is currently only known from a single location (Fig.&nbsp;2) where it was collected by leaf-litter extraction at the inner edge of a secondary forest.

### Strumigenys mazu

Taxon classificationAnimaliaHymenopteraFormicidae

(Terayama, Lin & Wu, 1996) – First recorded in Hong Kong in 2000 (Bolton 2000).

Smithistruma mazu Terayama Lin and Wu 1996: 337, figs 26, 27, 30, 31 (w.) TAIWAN. Indomalaya.
Pyramica mazu (Terayama, Lin & Wu, 1996). Combination in Pyramica: Bolton 1999: 1673.
Strumigenys mazu (Terayama, Lin & Wu, 1996). Combination in Strumigenys: Baroni Urbani and De Andrade 2007: 123.

#### Material examined.

HONG KONG: Sha Tin District, Tai Po Kau, 22.42007N, 114.1829E, 02.07.2015, 291 m, T. Tsang, Winkler, IBBL; Tsuen Wan District, Tai Lam Country Park, 22.38109N, 114.05511E, 8.XI.2017, 254 m, R. Cheung / M. Pierce, ANTWEB1016463, Winkler, IBBL.

#### Geographic range.

China (Guangxi), Hong Kong, Japan, Taiwan.

#### Ecology.

**Figure 10.**
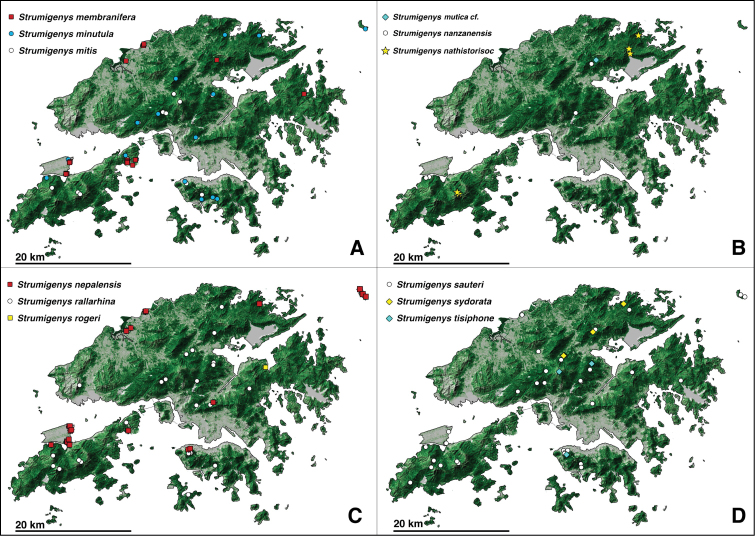
Distribution of *Strumigenys* species in Hong Kong **A***S.membranifera*, *S.minutula*, and *S.mitis***B**S.cf.mutica, *S.nanzanensis*, and *S.nathistorisoc* sp. n. **C***S.nepalensis*, *S.rallarhina*, and S.rogeri* **D***S.sauteri*, *S.sydorata*, and *S.tisiphone*. Circles represent species previously recorded from Hong Kong, diamonds represent newly recorded species, and stars represent new species. Newly recorded introduced species are shown with red squares. Green shaded portions of the map correspond with higher levels of tree cover, and grey with lower levels of tree cover. *To avoid confusion, *S.rogeri* (a newly recorded introduced species) is represented by a yellow square instead of a red square.

This is an uncommon species in Hong Kong where it is known only from a few locations (Fig. 10). This species occurs in secondary forests at elevations ranging from 262 to 291 m. It apparently forms small monogynous colonies of about 20 individuals (Masuko 2009b).

### Strumigenys membranifera

Taxon classificationAnimaliaHymenopteraFormicidae

Emery, 1869 - new record

Fig. 5D–F

Strumigenys (Trichoscapa) membranifera Emery 1869: 24, fig. 11 (w.) ITALY. Palearctic.
Strumigenys (Cephaloxys) membranifera (Emery, 1869). Combination in Strumigenys (Cephaloxys): Emery 1916: 205.
Trichoscapa membranifera (Emery, 1869). Combination in Trichoscapa: Brown 1948: 113.
Pyramica membranifera (Emery, 1869). Combination in Pyramica: Bolton 1999: 1673.
Strumigenys membranifera (Emery, 1869). Combination in Strumigenys: Baroni Urbani and De Andrade 2007: 123.
Senior synonym of S.foochowensis, S.membraniferamarioni, S.membraniferasantschii, S.silvestriana, S.membraniferasimillima, S.vitiensis, S.membraniferawilliamsi: Brown 1948: 114.

#### Material examined.

HONG KONG: Islands District, Chek Lap Kok, 22.2947N, 113.9336E, 21 m, B.M. Worthington, Winkler, 4 Corners, IBBL; Islands District, Chek Lap Kok, 22.2953N, 113.9354E, 28 m, B.M. Worthington, Winkler, 12 Random, IBBL; Islands District, Chek Lap Kok, 22.2953N, 113.9354E, B.M. Worthington, Winkler, 12 Random, IBBL; Islands District, Chek Lap Kok, 22.2957N, 113.9338E, 15 m, B.M. Worthington, Winkler, 12 Random, IBBL; Islands District, Chek Lap Kok, 22.2957N, 113.9338E, 15 m, B.M. Worthington, Winkler, 4 Corners, IBBL; Islands District, Chek Lap Kok, 22.2957N, 113.9338E, B.M. Worthington, Winkler, 12 Random, IBBL; Islands District, Chek Lap Kok, 22.3139N, 113.9398E, 2 m, B.M. Worthington, Winkler, 4 Corners, IBBL; Islands District, Chek Lap Kok, 22.3139N, 113.9398E, B.M. Worthington, Winkler, 12 Random, IBBL; Islands District, Chek Lap Kok, 22.3153N, 113.9407E, B.M. Worthington, Winkler, 12 Random, IBBL; Islands District, Chek Lap Kok, 22.3153N, 113.9407E, B.M. Worthington, Winkler, 4 Corners, IBBL; Islands District, Disneyland, 22.309433N, 114.04575E, 19.07.2016, B. Guénard, Gut content Water Dragon, IBBL; Tai Po District, Ping Shan Chai, 22.486N, 114.187E, 13.06.2015, C. Barthélémy, Malaise trap, IBBL; Tai Po District, To Kwa Peng, 22.42901N, 114.3336E, 25.05.2018, 1 m, R. Cheung / C. Taylor, Malaise trap, IBBL; Yuen Long District, Lok Ma Chau, 22.51031N, 114.06376E, 16.05.2018, 1 m, M. Wong, pitfall trap, IBBL; Yuen Long District, Lok Ma Chau, 22.51287N, 114.06463E, 17.07.2018, 1 m, M. Wong, pitfall trap, IBBL.

#### Measurements.

Workers (*n* = 5): TL 1.9–2.1, HL 0.45–0.47, HW 0.40–0.41, MandL 0.08–0.09, SL 0.20–0.23, EL 0.029–0.037, PW 0.22–0.24, ML 0.49–0.53, PL 0.22–0.26, PH 0.13–0.15, DPW 0.14–0.15, PPL 0.15–0.18, GL 0.47–0.53, CI 87–91, MI 17–20, SI 49–56, OI 7–9, LPI 55–58, DPI 54–61.

#### Geographic range.

*Native*: Ghana, Sierra Leone, South Africa

*Introduced*: a widespread species in multiple biogeographic realms. For a full global account, please refer to records presented under antmaps.org (Janicki et al. 2016, Guénard et al. 2017). Here, the Asian distribution is presented for the Oriental (China: Guangdong, Hong Kong, Yunnan; India; Taiwan) and Sino-Japanese realms (Bhutan, China: Fujian, Sichuan; Japan: Honshu, Kyushu, Ryukyu Islands, Shikoku; Nepal).

#### Ecology.

This is a species restricted to habitats with frequent disturbance, particularly within lowland areas (Hong Kong Airport, Disneyland, and Mai Po Nature Reserve) covered with grasslands or remnants of forests within urbanized matrices (Fig. 10). One alate female was collected in a Malaise trap between the June 13–27, suggesting potential swarming during this period in Hong Kong. Elevation ranged from 1 to 29 m, with the exception of an alate gyne collected at an elevation of 142 m. This species forms relatively large colonies of about 250 individuals (up to 350 individuals; Ito et al. 2010).

#### Comments.

The record of this tramp species in Hong Kong is not surprising considering its widespread range in the region and previous records in the nearby provinces of Guangdong and Fujian as well as from Macau for 90 years (Wheeler 1928), while extending further west in China to Yunnan. Considering the widespread range of this species in tropical and subtropical regions, it appears likely that this species is already present in two other provinces of China: Guangxi and Hainan. Future sampling efforts, in particular within urban habitats, could support this hypothesis.

### Strumigenys minutula

Taxon classificationAnimaliaHymenopteraFormicidae

Terayama & Kubota, 1989 – First recorded in Hong Kong in 2000 (Bolton 2000)

Strumigenys minutula Terayama and Kubota 1989: 782, figs 13–17 (w.q.) TAIWAN. Indomalaya.

#### Material examined.

HONG KONG: Central & Western District, Lung Fu Shan, 22.28221N, 114.133476E, 115 m, 13.11.2014, M. Wong, Winkler, 12 Random, IBBL; North District, H.W. Hang, 22.52819N, 114.2006E, 29 m, 14.06.2015, T. Tsang, Winkler, IBBL; North District, Lai Chi Wo, 22.527N, 114.258E, 08.05.2015, R.H. Lee, Winkler, IBBL; Sai Kung District, Pak Tam Chung, 22.454795N, 114.118215E, 05.06.2015, R.H. Lee, Winkler, IBBL; Sha Tin District, Tai Po Kau, 22.4198N, 114.1839E, 18.05.2016, B. Guénard, hand collection, IBBL; Southern District, Nam Fung Road, 22.25291N, 114.1877E, 70 m, 10.10.2015, T. Tsang, Winkler, IBBL; Southern District, Nam Fung Road, 22.25554N, 114.1802E, 01.10.2015, 110 m, T. Tsang, Winkler, IBBL; Tsuen Wan District, Lin Fa Shan, 22.3956N, 114.0885E, 15.07.2016, R.H. Lee, pitfall trap, IBBL.

#### Ecology.

This is a rather uncommon species collected from tree plantation, secondary forest, and Feng Shui woods (Fig. 10). The known elevation range of this species in Hong Kong is from 29 to 475 m. A colony of *S.minutula* including three dealate queens, 47 workers, 97 pupae, and 80 larvae were collected in a log at Tai Po Kau on May 18, 2016. The presence of multiple queens and the high number of pupae and larvae retrieved indicate that functional polygyny and large colony size (300 individuals) as previously reported occur within this species (Terayama et al. 2014). A single alate gyne was collected between June 26 and July 10 in a Malaise trap located within a mangrove area.

### Strumigenys mitis

Taxon classificationAnimaliaHymenopteraFormicidae

(Brown, 2000) – First recorded in Hong Kong in 1994 (Fellowes 1996)

Pyramica mitis Brown 2000: 442, figs 267, 290 (w.q.) PHILIPPINES. Indomalaya.
Strumigenys mitis (Brown, 2000). Combination in Strumigenys: Baroni Urbani and De Andrade 2007: 124.

#### Material examined.

HONG KONG: Central & Western District, Lung Fu Shan, 22.27896N, 114.13601E, 244 m, 20.11.2014, M. Wong, Winkler, 4 Corners, IBBL; Islands District, Sunset Peak, 22.26084N, 113.95753E, 03.06.2015, R.H. Lee, Winkler, IBBL; Islands District, Sunset Peak, 22.26392N, 113.95376E, 03.06.2015, R.H. Lee, Winkler, IBBL; Sha Tin District, Tai Po Kau, 22.42706N, 114.179996E, 06.06.2017, R.H. Lee, pitfall trap, IBBL; Southern District, Aberdeen Reservoir, 22.25964N, 114.16251E, 29.06.2015, R.H. Lee, Winkler, IBBL; Southern District, Aberdeen Reservoir, 22.26N, 114.162E, 26.06.2015, R.H. Lee, Winkler, IBBL; Southern District, Nam Fung Road, 22.25291N, 114.1877E, 10.10.2015, 70 m, T. Tsang, Winkler, IBBL; Tai Po District, Kadoorie Centre, 22.4291N, 114.11491E, 08.09.2015, R.H. Lee, Winkler, IBBL; Tsuen Wan District, Ha Lin Fa Shan, 22.39664N, 114.1019E, 30.07.2015, 355 m, T. Tsang, Winkler, IBBL; Tsuen Wan District, Ha Lin Fa Shan, 22.39854N, 114.0966E, 410 m, 18.07.2015, T. Tsang, Winkler, IBBL; Tsuen Wan District, Lin Fa Shan, 22.3956N, 114.0885E, 15.07.2016, R.H. Lee, Winkler, IBBL; Tsuen Wan District, Tai Mo Shan, 22.416073N, 114.125158E, 09.06.2015, R.H. Lee, pitfall trap, IBBL; Tsuen Wan District, Tai Mo Shan, 22.416073N, 114.125158E, 21.06.2016, R.H. Lee, pitfall trap, IBBL.

#### Ecology.

Although this species is not among the most commonly collected, it was found in a wide range of habitats and elevation, including grasslands, shrublands, tree plantations (e.g. *L.confertus*), and secondary forest at elevation ranging from 70 to 809 m (Fig. 10). Colonies apparently can be relatively small in size with about 50 individuals (Mezger and Pfeiffer 2008).

### Strumigenys cf. mutica

Taxon classificationAnimaliaHymenopteraFormicidae

(Brown, 1949) – New record

Fig. 11A–C

#### Material examined.

HONG KONG: Tai Po District, Ping Shan Chai, 22.486N, 114.187E, 142 m, 3.VI.2017 to 30.VI.2017, C. Barthélémy, ANTWEB1016246, Malaise trap, IBBL.

#### Measurements.

Alate females (*n* = 2): TL 2.2–2.5, HL 0.51–0.57, HW 0.39–0.41, MandL 0.13–0.14, SL 0.33–0.38, EL 0.14–0.16, PW 0.28–0.35, ML 0.58–0.68, PL 0.22–0.24, PH 0.19–0.20, DPW 0.13, PPL 0.11–0.13, GL 0.61–0.73, CI 72–76, MI 25, SI 85–92, OI 36–39, LPI 84–86, DPI 55–59.

**Figure 11.**
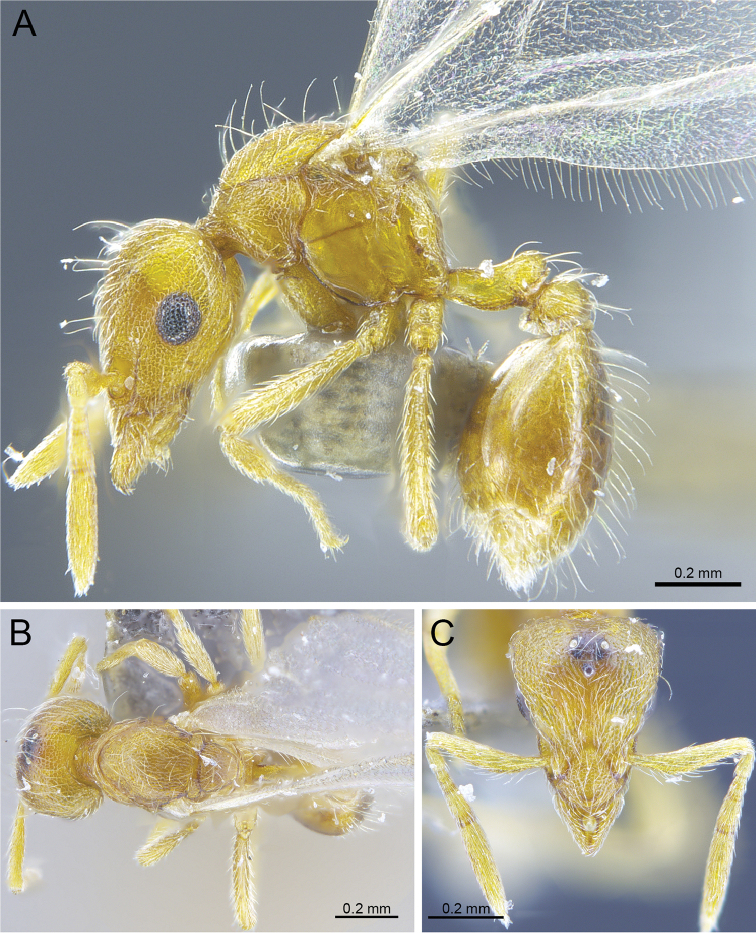
Strumigenyscf.mutica**A–C** queen (ANTWEB1016246(1)) **A** profile view **B** dorsal view **C**&nbsp;full-face view.

This species is known in Hong Kong from two alate females. The shape of the mandibles, including the conspicuous diastema and dentition suggests that this species belongs to the *S.mutica*-group as defined by Bolton (2000). However, this species differs from other Asian species in this group, defined on the basis of the worker caste, by the absence of spatulate or spoon-shaped hairs, instead having elongate fine hairs covering the body. However, the queen caste of *S.mutica* was originally described as a separate species, *Kyidrisnuda* (Brown 1949), but then synonymized with *S.mutica* on the basis of complete nest series reared in laboratory conditions (Brown 1952). The fine hairs on queens of *S.mutica* were described by Brown (1949) as short and pointed, which we confirmed after examination of photographs of the holotype of *K.nuda* (Japanese Ant Image Database 2006, pictures PCD2228–48, 49 & 50). This is contrary to our specimens, which possess long suberect and erect fine hairs (Fig. 11A–C). According to Brown (1949), queens of *S.mutica* also possess a densely punctulate-granulose mesonotal surface, while our specimens show a punctuate to finely strigate mesonotal surface, with shiny and smooth anepisternum and katepisternum. Unfortunately, we were not able to examine the queen specimen of *S.mutica* in great detail. While the specimens collected in Hong Kong might represent a new species within the *S.mutica*-group, we do not think that at this point enough evidence could be gathered to describe those as a new species. Future collection of workers or new available material of gynes collected in Taiwan or Japan might help solve this problem.

#### Ecology.

Very little is known about the ecology of *S.mutica*, as only two alate individuals collected in a secondary forest by Malaise traps are known (Fig. 10).

#### Comments.

Two species within the *S.mutica*-group have been recorded in nearby regions, *S.mutica* in mainland China (Guangxi, Hunan), Japan, South Korea, and Taiwan, and *S.takasago*, endemic to Taiwan. The latter species also differs from our specimens by its larger size (HL 0.70, HW 0.63), the conspicuous presence of erect spoon-shaped hairs on the body, and the acute propodeal declivity (Terayama et al. 1995).

### Strumigenys nanzanensis

Taxon classificationAnimaliaHymenopteraFormicidae

Lin & Wu, 1996 – First recorded in Hong Kong in 1993 (Fellowes 1996; Bolton 2000)

Strumigenys nanzanensis Lin and Wu 1996: 148, figs 13, 30–34 (w.q.) TAIWAN. Indomalaya.

#### Material examined.

HONG KONG: Tai Po District, Ping Shan Chai, 22.486N, 114.187E, 02.05.2015, C. Barthélémy, Malaise trap, IBBL; Tai Po District, Ping Shan Chai, 22.486N, 114.187E, 09.04.2016, C. Barthélémy, Malaise trap, IBBL; Tsuen Wan District, Shing Mun Reservoir, 22.39718N, 114.15273E, 230 m, 06.07.2011, P. Ward, sifted litter, IBBL.

#### Ecology.

This is a relatively rare species in Hong Kong known only from secondary forests at elevations between 143 and 230 m (Fig. 10).

### Strumigenys nathistorisoc sp. n.

Taxon classificationAnimaliaHymenopteraFormicidae

http://zoobank.org/039F77DA-9AE1-4293-94B2-10D1332FFD77

Fig. 12A–D

#### Diagnosis.

Dorsum of head, scape, and mandibles covered with appressed spatulate hairs, but no standing hairs. Side of mesosoma generally smooth and shining. Elongated propodeal spines subtended by narrow concave lamellae. Masticatory margin of mandibles engaging only at the apical half (or slightly less than half) of their lengths, with a prominent diastema proximal to this and first 3 preapical teeth not reaching their counterpart from the opposing mandible. Dentition consisting of a small conical tooth, a series of alternating long conical teeth and low round teeth, a crowded series of minute denticles at the down curvature of the apex of mandible, and a small conical apical tooth.

#### Type material.

Holotype worker: HONG KONG: Islands District, Lantau Island, Sunset Peak, 22.263923N, 113.953762E, 467 m, 3 June 2015 (R.H. Lee) (collection code RHL-HK-LSP-T3WM) [IBBL, ANTWEB1016948]. Paratype workers (*n* = 26): same data as holotype.

#### Non-type material examined.

Islands District, Sunset Peak, 22.26112N, 113.956332E, 572 m, 03.06.2015, R.H. Lee, Winkler, IBBL; North District, Lai Chi Wo, 22.527N, 114.258E, 08.05.2015, R.H. Lee, Winkler, IBBL; Tai Po District, Wu Kau Tang, 22.49645N, 114.2441E, 29 m, 25.10.2015, T. Tsang, Winkler, IBBL; Tai Po District, Wu Kau Tang, 22.5046N, 114.2422E, 115 m, 21.10.2015, T. Tsang, Winkler, IBBL.

#### Measurements.

Holotype worker: TL 3.2, HL 0.72, HW 0.57, MandL 0.24, SL 0.33, EL 0.068, PW 0.27, ML 0.79, PL 0.37, PH 0.15, DPW 0.15, PPL 0.26, GL 0.81, CI 79, MI 33, SI 58, OI 12, LPI 41, DPI 40. Paratype workers (*n* = 26): TL 2.9–3.3, HL 0.67–0.75, HW 0.51–0.60, MandL 0.22–0.25, SL 0.30–0.35, EL 0.064–0.080, PW 0.25–0.30, ML 0.75–0.86, PL 0.34–0.40, PH,0.15–0.18 DPW 0.14–0.17, PPL 0.22–0.26, GL 0.72–0.87, CI 76–81, MI 30–34, SI 56–62, OI 11–15, LPI 42–45, DPI 39–44.

#### Worker description.

**Figure 12.**
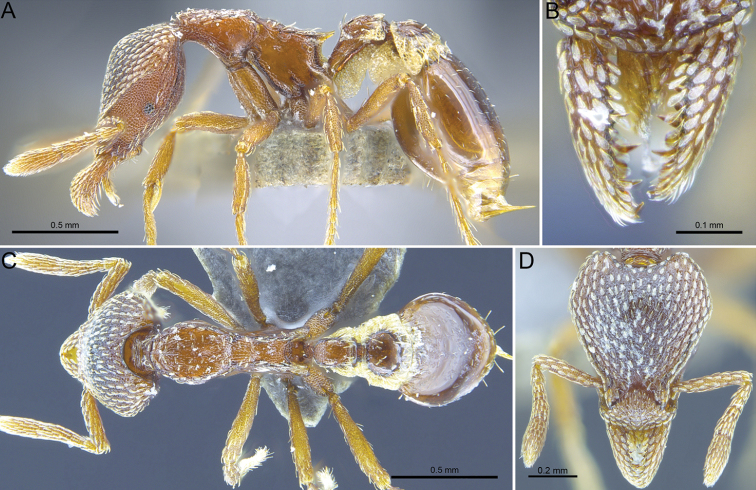
*Strumigenysnathistorisoc* sp. n. **A, C, D** Holotype worker (ANTWEB1016948), **B** paratype worker (ANTWEB1009636) **A** profile view **B** close-up on the mandibles in anterodorsal view **C** dorsal view **D** full-face view.

(Fig. 12A–D) ***Head.*** In full-face view occipital margin deeply, evenly concave; occipital corners well developed and bluntly angular. Clypeus, with a broadly convex anterior margin, in full-face view roughly resembling an inverted diamond-shape, broader than long; with a clear colour differentiation from the rest of the cephalic dorsum. Scapes subcylindrical, marginated but not converging anteriorly to form a thin lamella at leading edge. Mandibles in full-face view elongate and narrow, linear and very slightly curved; basal lamellae low and broadly triangular, not fully visible at full closure of the mandibles; in profile view robust with the apical half enlarged, portion following midpoint distinctively raised then curving downwards apically. Masticatory margins engage only at apical half (or slightly less than half) of their lengths; proximal to this is a prominent diastema between the mandibles, through which the labral lobes are visible.

***Dentition.*** Basal most preapical tooth small and conical, sometimes followed by a small denticle; the following tooth also conical, larger and longer; the third tooth low and rounded; in full-face view all first three teeth located on the basal half of the mandible and not reaching their counterpart from the opposing mandible when the mandibles are fully closed. Distal to these, the fourth tooth conical and slightly curved, being the longest of the preapical teeth; the fifth tooth low and rounded, sometimes almost squircle in shape, wider and longer than the third tooth; the sixth tooth conical, similar in length to second tooth; all these three teeth fully engaging with their counterparts from the opposing mandible and are visible in full-face view. Apex of mandible at the down curvature, in anterior view, with a crowded series of around 11 minute denticles, terminating with a small conical apical tooth.

***Mesosoma.*** In profile view the dorsum of mesosoma more or less flat transversely, except for a slight depression at the mesonotum immediately posterior to the pronotum; pronotum marginate dorsolaterally. In dorsal view, lateral margins of the pronotum evenly convex. Propodeum spines elongate acute triangular, subtended on each side by a very narrow concave lamella that broadens slightly basally into a small rounded convex propodeal lobe.

***Waist segments.*** Petiole in profile elongate and subclavate; peduncle does not grade evenly into the node, and about as long as (or slightly shorter than) the node; node in dorsal view longer than broad. Disc of the postpetiole in dorsal view broader than long, and slightly shorter than petiolar node. Spongiform tissues present on both petiole and postpetiole; ventral lobes of petiole and postpetiole extensive; lateral lobe of petiole merely a small flap at the posterolateral angle of the node in profile; in dorsal view present along the posterior margin of the petiolar node, and surrounding the disc of postpetiole, thicker along the posterior margin than that on the anterior margin.

***Pilosity.*** Cephalic dorsum in profile without standing hairs. In full-face view cephalic dorsum covered in rows of anteriorly directed appressed spatulate hairs that are slightly inclined toward the midline; no laterally projecting standing hair; cephalic dorsolateral margin, from the anterolateral margin of the occipital lobe to the frontal carina, with anteriorly directed appressed hairs; leading edge of scape with apically directed, appressed spatulate hairs, and an additional 2 or 3 rows of similar hairs on the surface of the scape. In full-face view, dorsal masticatory margin of mandibles with a row of anteriorly directed spatulate hairs that slightly inclined toward the midline; rest of dorsal surface of mandibles also densely covered in rows of similar hairs. Pronotal dorsum covered sparsely with appressed spatulate hairs directed toward the midline; row of similar appressed hairs on the dorsolateral margins of the pronotum. Propodeal dorsum with a few posteriorly directed, suberect to decumbent short simple hairs; a few similar hairs on the dorsolateral margin of the petiolar node and postpetiole. First gastral tergite with sparse, very short suberect simple hairs; in dorsal view with 1 or 2 pairs of simple apical erect hairs positioned laterally.

***Sculpture.*** Cephalic dorsum, excluding clypeal dorsum, sparsely reticulate-rugulose, with spaces between rugulae densely areolate-rugulose; clypeal dorsum densely reticulate-rugulose; surface of antennal scrobe, antenna and mandibles densely reticulate-punctate. Pronotal dorsum faintly reticulate-rugulose; mesonotal and propodeal dorsum densely reticulate-punctate; side of mesosoma generally smooth and shining, with vestiges of sculpture around margins. Dorsum of petiolar node faintly reticulate-punctate; dorsum of postpetiole smooth and shining. Basigastral costulae short and inconspicuous, rest of gaster smooth and shiny.

#### Comments.

*Strumigenysnathistorisoc* is well distinguished from other *Strumigenys* species by a combination of the characteristics listed in the diagnosis. It does not fit the description of any existing *Strumigenys* species group, in particular due to its highly distinctive mandibles and dentition.

Comparing *S.nathistorisoc* with the most similar appearing *Strumigenys* species, such as *S.kichijo* and species from the *S.leptothrix*-group, these species, in contrast, are often either hairless or have simple or flagellate hairs on the cephalic and pronotal dorsum, or have sculpture on the sides of the mesosoma, or have propodeal spines subtended by medium to broad lamellae. *S.nathistorisoc* also lacks the laterally projecting hairs in full-face view or the distinct transverse striations on the pronotal dorsum and dorsum of the petiolar node that characterise *S.nankunshana*.

Focusing on the mandibles and dentition, in contrast to the description of *S.nathistorisoc*, *S.kichijo* has only conical teeth. *S.nankunshana* and species from the *S.leptothrix*-group have masticatory margins that engage throughout the entire length. *S.wilsoniana* has a much wider gap between the mandibles, the masticatory margins engage at the apical third (instead of around half as in *S.nathistorisoc*) of their length, with the basal 2 teeth (instead of 3) situated at around the mid-length of the mandible (instead of sparsely across the basal half of the mandible) and not reaching their counterparts from the opposing mandible when the mandibles are fully closed.

#### Etymology.

This species is named after the Hong Kong Natural History Society whose members graciously supported our work on the ants of Hong Kong.

#### Geographic range.

Hong Kong

#### Ecology.

*Strumigenysnathistorisoc* was only recorded in secondary forests and Feng Shui woods, along a relatively large gradient of elevation ranging from 29 to 572 m (Fig. 10).

### Strumigenys nepalensis

Taxon classificationAnimaliaHymenopteraFormicidae

Baroni Urbani & De Andrade, 1994 - new record

Fig. 5G–I

Strumigenys nepalensis Baroni Urbani and De Andrade 1994: 57, figs 33, 34 (w.q.) NEPAL. Indomalaya.
Smithistruma nepalensis (Baroni Urbani & De Andrade, 1994). Combination in Smithistruma: Bolton 1995: 385.
Pyramica nepalensis (Baroni Urbani & De Andrade, 1994). Combination in Pyramica: Bolton 1999: 1673.
Strumigenys nepalensis Baroni Urbani & De Andrade, 1994. Combination in Strumigenys: Baroni Urbani and De Andrade 2007: 124.

#### Material examined.

HONG KONG: Central & Western District, HKU Campus, 22.282164N, 114.138296E, 113 m, 19.11.2014, M. Wong, Winkler, 12 Random, IBBL; Central & Western District, HKU CYT, 22.2824528N, 114.14043E, 07.01.2016, G. Yong, Winkler, IBBL; Islands District, Chek Lap Kok, 22.2939N, 113.9331E, B.M. Worthington, Winkler, 12 Random, IBBL; Islands District, Chek Lap Kok, 22.2947N, 113.9336E, 21 m, B.M. Worthington, Winkler, 4 Corners, IBBL; Islands District, Chek Lap Kok, 22.2957N, 113.9338E, B.M. Worthington, Winkler, 4 Corners, IBBL; Islands District, Chek Lap Kok, 22.2964N, 113.9352E, 10 m, B.M. Worthington, Winkler, 4 Corners, IBBL; Islands District, Chek Lap Kok, 22.298N, 113.9359E, 5 m, B.M. Worthington, Winkler, 4 Corners, IBBL; Islands District, Chek Lap Kok, 22.298N, 113.9359E, B.M. Worthington, Winkler, 4 Corners, IBBL; Islands District, Chek Lap Kok, 22.3139N, 113.9398E, 2 m, B.M. Worthington, Winkler, 4 Corners, IBBL; Islands District, Chek Lap Kok, 22.3153N, 113.9407E, B.M. Worthington, Winkler, 12 Random, IBBL; Islands District, Chek Lap Kok, 22.3204N, 113.9376E, 6 m, B.M. Worthington, Winkler, 12 Random, IBBL; Islands District, Chek Lap Kok, 22.3193N, 113.9377E, B.M. Worthington, Winkler, 12 Random, IBBL; Islands District, Sha Lo Wan, 22.2898N, 113.9069E, 43 m, B.M. Worthington, Winkler, 4 Corners, IBBL; Islands District, Tung Chung, 22.2907N, 113.9371E, 1 m, B.M. Worthington, Winkler, 4 Corners, IBBL; North District, Lai Chi Wo, 22.527N, 114.258E, 08.05.2015, R.H. Lee, Winkler, IBBL; Sha Tin District, Lion Rock, 22.360915N, 114.180028E, 13.07.2015, R.H. Lee, Winkler, IBBL; Yuen Long District, Mai Po, 22.48121N, 114.03332E, 30.07.2018, 1 m, M. Wong, pitfall trap, IBBL; Yuen Long District, Mai Po, 22.48625N, 114.04097E, 03.08.2018, 1 m, M. Wong, pitfall trap, IBBL. MACAU: Hac Sa Reservoir, Coloane Island, 22.1344N, 113.5725E, 20.08.2016, 93 m, C.M. Leong, Winkler, IBBL.

#### Measurements.

Workers (*n* = 8): TL 1.3–1.8, HL 0.42–0.46, HW 0.31–0.33, MandL 0.08–0.09, SL 0.18–0.20, EL 0.033–0.043, PW 0.19–0.21, ML 0.44–0.48, PL 0.20–0.22, PH 0.11–0.13, DPW 0.10–0.11, PPL 0.13–0.15, GL 0.43–0.47, CI 70–76, MI 17–21, SI 56–63, OI 11–13, LPI 52–58, DPI 48–54.

#### Geographic range.

China (Yunnan), India, Malaysia, Nepal, Singapore, Thailand, Vietnam.

***Introduced***: Mauritius, Hong Kong.

#### Ecology.

This is a common species in urban forest patches or disturbed grassland (e.g. Mai Po Nature Reserve), with only a few records collected within secondary forests and one record within Feng Shui woods (Fig. 10). Elevation records ranged from 1 to 135 m, suggesting that this species might prefer lowland habitats. The association of this species with relatively disturbed habitats suggests a potential tramp species, although other biological characteristics (e.g. polygyny, unicoloniality) are unknown at present.

#### Comments.

New records of *Strumigenysnepalensis* in Hong Kong expand the current known native range of this species by 800 km eastward from Vietnam. A record from Mauritius (Casent0799280, Ile Aux Aigrettes, −20.419017, 57.730183, 5 m a.s.l., A. Suarez 2.VI.2005; Doug Booher pers. comm.) confirms the tramp character of this species. Specimens collected from Hong Kong, Macau and Mauritius are considered introduced.

### Strumigenys rallarhina

Taxon classificationAnimaliaHymenopteraFormicidae

Bolton, 2000 – First record in Hong Kong in 1978 (Bolton 2000)

Strumigenys rallarhina Bolton 2000: 891 (w.) CHINA. Palearctic.

#### Material examined.

HONG KONG: Central & Western District, Lung Fu Shan Park, 22.280693N, 114.137027E, 3 Oct. 2018, B. Guénard, hand collection, IBBL; Central & Western District, Lung Fu Shan, 22.2751778N, 114.138576E, 07.01.2016, G. Yong, Winkler, IBBL; Central & Western District, The Peak, 22.276038N, 114.141995E, 17.08.2015, R.H. Lee, Winkler, IBBL; Islands District, Lamma Island, 22.20575N, 114.13808E, 14.09.2017, R.H. Lee, Winkler, IBBL; Islands District, Lantau Peak, 22.249N, 113.921E, 15.09.2015, R.H. Lee, pitfall trap, IBBL; Islands District, Sunset Peak, 22.260842N, 113.957533E, 03.06.2015, R.H. Lee, Winkler, IBBL; Islands District, Sunset Peak, 22.263923N, 113.953762E, 03.06.2015, R.H. Lee, pitfall trap, IBBL; Islands District, Sunset Peak, 22.263923N, 113.953762E, 03.06.2015, R.H. Lee, Winkler, IBBL; Sha Tin District, Lion Rock, 22.357002N, 114.175047E, 13.07.2015, R.H. Lee, Winkler, IBBL; Sha Tin District, Lion Rock, 22.36121N, 114.181997E, 15.08.2017, R.H. Lee, Winkler, IBBL; Sha Tin District, Mau Ping Wood, 22.3844N, 114.241E, 20.10.2015, R.H. Lee, Winkler, IBBL; Sha Tin District, Tai Po Kau Nature Reserve, 22.4281N, 114.1808E, 24.02.2016, B. Guénard, Winkler, IBBL; Sha Tin District, Tai Po Kau, 22.42402N, 114.18029E, 14.07.2015, R.H. Lee, Winkler, IBBL; Southern District, Nam Fung Road, 22.2546N, 114.1833E, 20.08.2016, R.H. Lee, pitfall trap, IBBL; Tai Po District, Sha Lo Tong, 22.47708N, 114.18195E, 28.05.2015, R.H. Lee, Winkler, IBBL; Tai Po District, Sha Lo Tong, 22.47808N, 114.18193E, 28.05.2015, R.H. Lee, Winkler, IBBL; Tai Po District, Sha Shan, 22.449N, 114.145E, 03.11.2015, R.H. Lee, Winkler, IBBL; Tsuen Wan District, Shing Mun Reservoir, 22.39718N, 114.15273E, 230 m, 06.07.2011, P. Ward, sifted litter, IBBL; Tai Po District, Tai Om, 22.442321N, 114.134738E, 28.02.2018, B. Guénard, Winkler, IBBL; Tsuen Wan District, Shing Mun, 22.39678N, 114.1531E, 238 m, 23.08.2015, T. Tsang, Winkler, IBBL; Tsuen Wan District, Shing Mun, 22.39693N, 114.153E, 14.05.2015, R.H. Lee, Winkler, IBBL; Tsuen Wan District, Tai Lam, 22.3956N, 114.0928E, 26.10.2015, R.H. Lee, Winkler, IBBL; Tuen Mun District, Castle Peak, 22.389935N, 113.954937E, 30.06.2015, R.H. Lee, Winkler, IBBL.

#### Ecology.

This is a relatively widespread species collected in a wide range of habitats and elevation, including grassland, roadside trees, shrubland, bamboo forest, secondary forest and Feng Shui woods at elevation ranging from 56 to 589 m (Fig. 10).

### Strumigenys rogeri

Taxon classificationAnimaliaHymenopteraFormicidae

Emery, 1890 - new record

Fig. 5J–L

Strumigenys rogeri Emery 1890: 68, pl. 7, fig. 6. SAINT THOMAS, U.S. VIRGIN ISLANDS. Neotropical.

#### Material examined.

HONG KONG: Tai Po District, Sai Keng, 22.41998N 114.26824E, 1 m, 26.VI.2018–10.VII.2018, R. Cheung / C. Taylor, Malaise trap, IBBL. VIETNAM: Cat Tien National Park, 11.26.237N, 107.25.431E, 145 m, 3.VI.2018, B. Guénard, IBBL, hand collection.

#### Measurements.

Alate (*n* = 1) TL 2.5, HL 0.58, HW 0.46., MandL 0.32, SL 0.35, EL 0.10, PW 0.32, ML 0.60, PL 0.28, PH 0.15, DPW 0.13, PPL 0.10, GL 0.62, CI 78, MI 54, SI 77, OI 21, LPI 54, DPI 48.

#### Geographic range.

***Native***: Afrotropical region, known from Ivory Coast to Zanzibar Archipelago (Tanzania) and south to Angola.

***Introduced***: A widespread species in multiple biogeographic realms. For a full global account, see antmaps.org (Janicki et al. 2016; Guénard et al. 2017). Here, the Asian distribution is presented for the Oriental realm (Hong Kong; Kerala [India]; Java and Sumatra [Indonesia], Peninsular and East Malaysia [Malaysia], Philippines, and Taiwan). We can also confirm the presence of this species from Vietnam, which was previously reported by Zryanin (2011).

#### Comments.

The record of this tramp species in Hong Kong is not surprising considering its widespread range in nearby countries (Philippines, Taiwan, and Vietnam), which have relatively similar climatic conditions. However, a single alate has been collected from a mangrove habitat, an unlikely habitat for this species, and no workers have been collected in Hong Kong. Nonetheless, the record from Hong Kong is the first observation of this species for mainland China.

### Strumigenys sauteri

Taxon classificationAnimaliaHymenopteraFormicidae

(Forel, 1912) – First record in Hong Kong in 1994 (Fellowes 1996)

Pentastruma sauteri Forel 1912: 51 (w.) TAIWAN. Indomalaya.
Pyramica sauteri (Forel, 1912). Combination in Pyramica: Bolton 1999: 1673.
Strumigenys sauteri (Forel, 1912). Combination in Strumigenys: Baroni Urbani and De Andrade 2007: 127.

#### Material examined.

HONG KONG: Central & Western District, HKU CYT, 22.2824528N, 114.140431E, 07.01.2016, G. Yong, Winkler, IBBL; Central & Western District, LFS Plot 1 B–C, 22.277134N, 114.134793E, 28.12.2015, G. Yong, Winkler, IBBL; Central & Western District, LFS Plot 1 C, 22.277134N, 114.134808E, 28.12.2015, G. Yong, Winkler, IBBL; Central & Western District, Lung Fu Shan, 22.276729N, 114.136693E, 295 m, 24.11.2014, M. Wong, Winkler, 4 Corners, IBBL; Central & Western District, Lung Fu Shan, 22.27896N, 114.13601E, 244 m,20.11.2014, M. Wong, Winkler, 12 Random, IBBL; Central & Western District, Lung Fu Shan, 22.28221N, 114.133476E, 115 m, 13.11.2014, M. Wong, Winkler, 12 Random, IBBL; Central & Western District, Plot 1 B–C, 22.277134N, 114.134794E, 08.01.2016, G. Yong, Winkler, IBBL; Islands District, Sunset Peak, 22.26112N, 113.956332E, 03.06.2015, R.H. Lee, Winkler, IBBL; Islands District, Tei Tong Tsai, 22.257066N, 113.926281747627E, 29.11.2016, R.H. Lee, Winkler, IBBL; North District, A Ma Wat, 22.5191N, 114.2441E, 19.12.2016, R.H. Lee, Winkler, IBBL; Sai Kung District, Pak Tam Chung, 22.400033N, 114.330997E, 05.06.2015, R.H. Lee, pitfall trap, IBBL; Sha Tin District, Lion Rock, 22.36121N, 114.181997E, 13.07.2015, R.H. Lee, pitfall trap, IBBL; Sha Tin District, Tai Po Kau Nature Reserve, 22.4288N, 114.1813E, 22.02.2017, B. Guénard, Winkler, IBBL; Sha Tin District, Tai Po Kau, 22.41841N, 114.1779E, 295 m, 12.07.2015, T. Tsang, Winkler, IBBL; Sha Tin District, Tai Po Kau, 22.427285N, 114.181298E, 16.09.2015, B. Guénard, hand collection, IBBL; Sha Tin District, Tai Po Kau, 22.42781N, 114.181462E, 08.10.2018, B. Guénard, hand collection, IBBL; Southern District, Aberdeen Reservoir, 22.259638N, 114.162508E, 29.06.2015, R.H. Lee, Winkler, IBBL; Southern District, Aberdeen Reservoir, 22.26N, 114.162E, 26.06.2015, R.H. Lee, Winkler, IBBL; Tai Po District, Hunch Backs, 22.4139N, 114.2489E, 13.11.2015, R.H. Lee, pitfall trap, IBBL; Tai Po District, Ping Shan Chai, 22.486N, 114.187E, 19.03.2016, C. Barthélémy, Malaise trap, IBBL; Tai Po District, Tai Om, 22.44157N, 114.13513E, 28.02.2018, B. Guénard, Winkler, IBBL; Tai Po District, Tap Mun, 22.47N, 114.363E, 28.07.2015, R.H. Lee, pitfall trap, IBBL; Tsuen Wan District, Ha Lin Fa Shan, 22.39608N, 114.1014E, 28.07.2015, 344 m, T. Tsang, Winkler, IBBL; Tsuen Wan District, Lin Fa Shan, 22.3956N, 114.0885E, 15.07.2016, R.H. Lee, pitfall trap, IBBL; Tsuen Wan District, Lin Fa Shan, 22.3956N, 114.0885E, 15.07.2016, R.H. Lee, Winkler, IBBL; Tsuen Wan District, Shing Mun, 22.39845N, 114.1628E, 367 m, 24.08.2015, T. Tsang, Winkler, IBBL; Tsuen Wan District, Shing Mun, 22.39962N, 114.162E, 355 m, 12.08.2015, T. Tsang, Winkler, IBBL; Yuen Long District, Kap Lung, 22.41596N, 114.1038E, 288 m, 11.09.2015, T. Tsang, Winkler, IBBL; Yuen Long District, Ng Tung Chai 22.43492N, 114.12927E, 01.11.2016, R.H. Lee, Winkler, IBBL.

#### Ecology.

This is a widespread species found in diverse habitats including grasslands, shrublands, plantations (e.g. *L.confertus*), urban forest remnants, secondary forest, and Feng Shui woods. Specimens were collected at elevation ranging from 19 to 630 m (Fig. 10).

### Strumigenys sydorata

Taxon classificationAnimaliaHymenopteraFormicidae

Bolton, 2000 - new record

Fig. 8D–F

Strumigenys sydorata Bolton 2000: 876 (w. q.) JAVA. Indomalaya.

#### Material examined.

HONG KONG: North District, Kuk Po San Uk, 22.52912N, 114.23468E, 15.11.2016, R.H. Lee, Winkler, IBBL; Sha Tin District, Tai Po Kau, 22.42614N, 114.18178E, 162 m, 06.07.2017, R.H. Lee, pitfall trap, IBBL; Tai Po District, Sha Lo Tong, 22.481767N, 114.18283E, 28.05.2015, R.H. Lee, Winkler, IBBL; Tai Po District, Tai Om, 22.4419N, 114.1335E, 76 m, 05.10.2016, Winkler, IBBL; Tai Po District, Tai Om, 22.4423N, 114.1343E, 81 m, 07.08.2015, T. Tsang, Winkler, IBBL.

#### Measurements.

Worker (*n* = 1): TL 2.5, HL 0.69, HW 0.53, MandL 0.19, SL 0.28, EL 0.059, PW 0.30, ML 0.66, PL 0.24, PH 0.17, DPW 0.15, PPL 0.16, GL 0.52, CI 77, MI 28, SI 53, OI 11, LPI 70, DPI 62.

#### Geographic range.

China (Hong Kong), Indonesia (Java), Thailand, Vietnam.

#### Ecology.

This is a rare species in Hong Kong collected only within secondary forests and Feng Shui woods (Fig. 10). Elevations of collection sites ranged from 15 to 170 m.

#### Comments.

This new record from Hong Kong represents another important geographic extension of 900 km north-eastward in Mainland Asia, with the closest record known from Cúc Phương in Vietnam (Eguchi et al. 2011). *Strumigenyssydorata* belongs to the *lyroessa*-complex within the *S.lyroessa*-group. This species can be separated from others in this group by the presence of pronotal humeral hairs, a smooth first gastral tergite, a well-developed lamella along the propodeal declivity, and a larger preapical tooth when compared to the apicodorsal tooth on mandibles. The latter character separates it from *S.arrogantia*, which is slightly smaller than *S.sydorata*.

### Strumigenys tisiphone

Taxon classificationAnimaliaHymenopteraFormicidae

Bolton, 2000 - new record

Fig. 8G–I

Pyramica tisiphone Bolton 2000: 390 (w.) CHINA. Indomalaya.
Strumigenys tisiphone (Bolton, 2000). Combination in Strumigenys: Baroni Urbani and De Andrade 2007: 129.

#### Material examined.

HONG KONG: Central & Western District, Lung Fu Shan, 22.27518N, 114.13858E, 07.01.2016, G. Wong, Winkler, IBBL; Sha Tin District, Tai Po Kau Nature Reserve, 22.4288N, 114.1813E, 22.02.2017, B. Guénard, Winkler, IBBL; Tsuen Wan District, Tai Mo Shan, 22.41496N, 114.12608E, 816 m, 24.06.2016, R.H. Lee, Winkler, IBBL.

#### Measurements.

Worker (*n* = 1): TL 2.4, HL 0.54, HW 0.50, MandL 0.20, SL 0.24, EL 0.036, PW 0.29, ML 0.59, PL 0.29, PH 0.16, DPW 0.16, PPL 0.19, GL 0.55, CI 93, MI 37, SI 48, OI 7, LPI 56, DPI 56.

#### Geographic range.

Hong Kong, Guangdong, Hubei, Hunan (China).

#### Ecology.

This is a rare species in Hong Kong, collected only within secondary forest but through a wide elevational range extending from 141 to 816 m (Fig. 10).

#### Comments.

The Hong Kong record confirms the distribution of *S.tisiphone* within China and represents the south-easternmost record for the species. The previous record in China is from Gutian, central Guangdong (24.2N, 116.6E) (Bolton 2000).

## Discussion

The genus *Strumigenys* currently includes 839 valid species and ranks as the third most diverse ant genus, after *Camponotus* (1031 valid species + 457 subspecies) and *Pheidole* (1004 valid species + 134 subspecies) (Bolton 2018). However, despite its hyperdiverse status, our results suggest that even 70 years after its publication, the statement by William Brown Jr (1949: 1) that “…the dacetine ants presently known from eastern Asia undoubtedly represent only a fraction of the species which actually exist there…” might still apply. As Hong Kong is a small territory of 1100 km² with a history of extensive deforestation and disturbance over nearly all of its territory (Zhuang and Corlett 1997), the discovery of three new species and nine new records (Table 2) stresses the need for further sampling and taxonomic work on this genus within southeastern China. These results show that even within a region with high disturbance history, and thus usually perceived as of lower ecological quality, taxonomic knowledge on a particular group of insect is still highly fragmented. Undoubtedly, future myrmecological surveys in this region will lead to the discovery of new species and the collection of new records. For instance, species such as *S.minutula* and *S.sydorata*, for which the new records presented here represent a disjunction within south-east China, or other widely distributed species in Hong Kong such as *S.canina*, *S.feae*, *S.hirsuta*, *S.heteropha*, *S.nathistorisoc*, *S.rallarhina*, and *S.sauteri*, are expected to be found in the nearby provinces of Guangdong, Guangxi, and Hainan (Hainan is currently devoid of any *Strumigenys* records [antmaps.org, November 2018]). With the addition of the three newly described species here, the total number of *Strumigenys* species known only from Hong Kong is now four, including *S.heteropha*, described by Bolton (2000) and widespread in Hong Kong (Fig. 10). However, this apparent endemism is likely the result of a lack of sampling in south-eastern China rather than a true biogeographic pattern. However, as urban development and deforestation within south-east China is expanding, the populations of these species in Hong Kong might become increasingly isolated. As such, the evolution of these populations and their conservation, coupled with potential limited dispersal abilities, might represent a good study system to address questions related to large scale fragmentation, genetic drift, and species or population extinction.

**Table 2.** List of the twenty-four *Strumigenys* species recorded in Hong Kong, with reference of their first record, collection within recent years (IBBL = Insect Biodiversity and Biogeography Laboratory at HKU) and type of habitat collected presented.

*Strumigenys* species	First published record in HK	Specimen at IBBL	Type of habitat
*S.canina* Brown & Boisvert, 1979	Fellowes 1996	Yes	Secondary forest; tree plantation; Feng Shui woods
*S.elegantula* Terayama & Kubota, 1989	Fellowes 1996	Yes	Reclaimed land; mixed woodland; semi-open forest
*S.emmae* Emery, 1890	Fellowes 1999	Yes	Reclaimed land; secondary forest
*S.exilirhina* Bolton, 2000	Bolton 2000	Yes	Semi-open forest; secondary forest; reclaimed land
*S.feae* Forel, 1912	Bolton 2000	Yes	Disturbed secondary forest
*S.formosa* Terayama. Ling & Wu, 1995	New record	Yes	Secondary forest
*S.heteropha* Bolton, 2000	Bolton 2000	Yes	Semi-open forest
*S.hexamera* Brown, 1958	New record	Yes	Secondary forest
*S.hirsuta* sp. n.	New species	Yes	Disturbed secondary forest; semi-open forest
*S.kichijo* Terayama, Lin & Wu, 1996	New record	Yes	Secondary forest
*S.lantaui* sp. n.	New species	Yes	Reclaimed land
*S.mazu* Terayama, Lin & Wu, 1996	Bolton 2000	Yes	Reclaimed land
*S.membranifera* Emery, 1869	New record	Yes	Secondary forest
*S.minutula* Terayama & Kubota, 1989	Bolton 2000	Yes	Semi-open forest; reclaimed land
*S.mitis* Brown, 2000	Fellowes 1996	Yes	Disturbed secondary forest; semi-open forest
S.cf.mutica (Brown, 1949)	New record	Yes	Mangrove (alates in Malaise trap)
*S.nanzanensis* Lin & Wu, 1996	Fellowes 1996	Yes	Secondary forest
*S.nathistorisoc* sp. n.	New species	Yes	Secondary forest
*S.nepalensis* Baroni Urbani & De Andrade, 1994	New record	Yes	Secondary forest
*S.rallarhina* Bolton, 2000	Bolton 2000	Yes	Secondary forest; semi-open forest; Feng Shui woods
*S.rogeri* Emery, 1890	New record	Yes	Mangrove (alate in Malaise trap)
*S.sauteri* Forel, 1912	Fellowes 1996	Yes	Semi-open forest; secondary forest
*S.sydorata* Bolton, 2000	New record	Yes	Feng Shui woods
*S.tisiphone* Bolton, 2000	New record	Yes	Secondary forest

The number of native *Strumigenys* species now recorded from continental China is 49 (Guénard et al. 2012, 2017), nearly half of which are found in Hong Kong (19 native species). With 24 species recorded (Table 1), the *Strumigenys* fauna of Hong Kong can be considered especially diverse for the region. In comparison, only 9, 13, and 17 native species have been recorded in the southern provinces of Guangdong, Guangxi, and Yunnan, respectively. Again, this suggests insufficient sampling. In contrast, 30 species are known from Taiwan, which has a much longer history of survey and taxonomic work (e.g. Terayama and Kubota 1989; Terayama 2009). While a multitude of sampling approaches have been deployed across Hong Kong over the past 5 years, the use of Winkler extractors in particular has allowed the collection of numerous *Strumigenys* specimens. As a result, the large increase in new species and records match those of a previous study conducted in Yunnan, which recorded six additional *Strumigenys* species for the province on the basis of a 3-week survey (Liu et al. 2015). Because the use of sampling methods specifically targeting leaf-litter ants has been seldom used in China and other Asian countries, we recommend a more systematic and generalized use of these methods to survey the local myrmecofauna.

**Figure 13.**
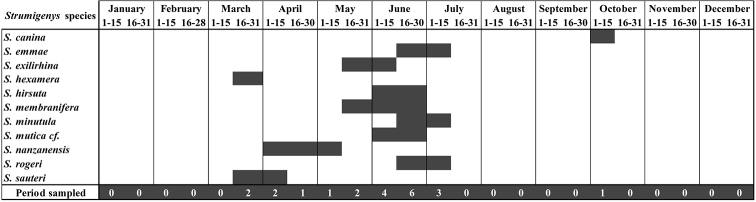
Phenology of 11 *Strumigenys* species collected in Hong Kong on the basis of alate female specimens collected within Malaise traps, with the exception of *S.canina* with females and males collected in leaf litter. Dark grey areas represent periods during which a given species was collected (in front of a species name) or the period in which sampling was conducted (Period sampled). Numbers in the last row of the table represent the number of species collected within a given period.

In addition, the use of Malaise traps resulted in the discovery of new species records on the basis of alate gynes (S.cf.mutica and *S.rogeri*). This resulted in new information on the phenology of several *Strumigenys* species within Hong Kong. While many tropical ant species exhibit multiple swarming periods (Kaspari et al. 2001), our results, though preliminary, indicate that each individual species’ nuptial flight is restricted to a period of a few days to a few weeks, with no instance of multiple distinct nuptial flight periods recorded. It is also interesting to note that 10 of the species collected were caught in a period ranging over only 4 months between late-March to early-July (Fig. 13). This might indicate that most species of *Strumigenys* in Hong Kong use a relatively short period of the year characterised by warmer temperature (21–28 °C) and heavier precipitation, with a peak of precipitation observed in June (Hong Kong Observatory 2018) corresponding to the period during which a maximum of species were observed swarming. The only exception to this was *S.canina*, whose alates, including 24 females, 1 female pupa, and 1 male, were collected in early October from leaf litter, potentially indicating swarming in the later part of the year characterised by drier weather conditions. Surprisingly, while *S.canina* was one of the most commonly encountered species in Hong Kong, we were unable to detect any alate females using Malaise traps. *Strumigenys* female alates were rather uncommon in Malaise traps, with only a handful of individuals collected from several hundred Malaise trap samples. This might indicate either that *Strumigenys* females are poor fliers or that Malaise traps installed above ground (about 1.8–2 m high) are not appropriate for capturing them. Finally, while male *Strumigenys* could be identified to the genus level and seemed more abundant than females, as observed in previous studies (Feitosa et al. 2016), we could not associate them with a particular species; this limited the information that we could retrieve. Thus, in order to understand the phenology of *Strumigenys* in the region, taxonomic classification of males should be a priority for future studies.

By its central position in Asia and its leading role in regional and global trade, Hong Kong presents numerous opportunities for the introduction, establishment, and spread of introduced species (Lu et al. 2018). Our results suggest that introduced *Strumigenys* species are particularly diverse and common in open and disturbed habitats. Prior to this study, only *S.emmae* had been recorded in the mid-1990s (Fellowes 1999) and more recently in multiple instances in urban environments of Hong Kong as well as in the neighbouring region of Macau (Leong et al. 2017). Future surveys within the Pearl River Delta Metropolitan Region will probably reveal a more widespread distribution of this species within urban habitats. More significantly, our results indicate an increase in introduced species since the survey performed 20 years ago (Fellowes 1996, 1999), with four species newly recorded from Hong Kong: *S.hexamera*, *S.membranifera*, *S.nepalensis*, and *S.rogeri*. These results, in combination with recent publications, confirm the spread of several introduced *Strumigenys* species throughout Southeast Asia. For instance, the introduced species *S.eggersi* has recently been recorded in the Philippines (General 2018) and Singapore (Wang and Yamane 2017). The establishment of this species in Hong Kong is plausible considering its presence as an introduced species within regions with similar climatic conditions as those observed in Hong Kong (e.g. Florida). Moreover, two species recorded in our study, *S.emmae* and *S.membranifera*, were also recently recorded for the first time from Sri Lanka (Dias et al. 2018). While distinguishing the recent spread of these species from the effects of increased sampling effort targeting anthropogenic habitats is impossible with the data currently available, it nonetheless shows that introduced *Strumigenys* species are probably more widespread than previously described. On the other hand, records for another species, *S.silvestrii*, recorded from Macau (Hua 2006), would require confirmation, as the origin of this record is uncertain and recent myrmecological work conducted in Macau, although limited in scope, failed to collect this species (Leong et al. 2017). Hence, this record might either be a misinterpretation of a record of *Strumigenyssilvestriana* (a synonym of *Strumigenysmembranifera*) from Macau in Chapman and Capco (1951); or, given that *S.silvestrii* has been recorded from both Portugal and Madeira islands (MacGown et al. 2012), and Macau used to be a Portuguese territory, an indication that this species was introduced there through the intense trade during the colonial period.

Globally, 24 *Strumigenys* species have been recorded outside of their putative native range (Table 3), with two species without established populations but intercepted during quarantine process. The largest number of introduced *Strumigenys* species is recorded within the Nearctic realm (11), with Florida alone hosting 10 introduced species (Deyrup 2016), followed by the Malagasy (8), and the Oceanian and Panamanian (7) realms. If five *Strumigenys* species have been introduced to the Oriental realm, a similar number of species originating from this realm have been introduced to other parts of the world, while the Sino-Japanese realm acts more as an exporter of *Strumigenys* species (4 species) than as an importing realm (2 species). Several species with their native range in Japan or China (e.g. *S.hexamera*, *S.lewisi*) have been recorded in Europe (Schembri and Collingwood 1995) and in the U.S.A. (Deyrup and Cover 2009). However, for several species, these records represent non-established populations detected during quarantine inspections (e.g. *S.minutula* [Boer and Vierbergen 2008], *S.solifontis* [Brown 1949]). If none of the introduced *Strumigenys* species are suspected to reach an invasive level, the ecological traits of these species (specialized predators found within leaf litter) challenge the general paradigm of many generalist introduced ant species. As a result, the genus represents an interesting study system to understand introduction mechanisms that favour the establishment of particular species within new regions, and to study their potential impacts or roles in their introduced range. Finally, local myrmecologists, particularly within Asia, are encouraged to conduct inventory within urban areas (parks, university campus, around airports, or ports) to detect potential new records of introduced *Strumigenys* species, as the introduced species detected in this study are likely to have widespread distributions within the region.

**Table 3.** List of the 24 *Strumigenys* species with records outside their native range with a presentation by biogeographic realms of their putative native and introduced ranges (data from antmaps.org, Janicki et al. 2016). Definitions of biogeographic realms follow Holt et al. 2013.

*Strumigenys* species	Native range	Introduced range
*S.eggersi* Emery, 1890	Neotropical, Panamanian	Nearctic, Oriental, Panamanian (Galapagos Islands)
*S.emmae* (Emery, 1890)	Australian	Afrotropical, Madagascan, Nearctic, Neotropical, Oceanian, Oriental, Panamanian, Saharo-Arabian
*S.epinotalis* Weber, 1934	Neotropical, Panamanian	Nearctic
*S.godeffroyi* Mayr, 1866	Australian, Oceanian (West part), Oriental	Madagascan, Oceanian (East part),
*S.gundlachi* (Roger, 1862)	Neotropical, Panamanian	Nearctic
*S.hexamera* (Brown, 1958)	Oriental, Sino-Japanese	Nearctic, Oceanian
*S.lanuginosa* Wheeler, 1905	Neotropical, Panamanian	Nearctic
*S.lewisi* Cameron, 1886	Oriental, Sino-Japanese	Oceanian, Palearctic (West)
*S.louisianae* Roger, 1863	Nearctic, Neotropical, Panamanian	Panamanian (Cocos Island, Galapagos Islands)
*S.ludovici* Forel, 1904	Afrotropical	Madagascan
*S.lujae* Forel, 1902	Afrotropical	Oceanian (not established)
*S.mandibularis* Smith, 1860	Neotropical	Afrotropical, Madagascan
*S.margaritae* Forel, 1893	Nearctic (south), Neotropical, Panamanian	Nearctic (north)
*S.maxillaris* Baroni Urbani, 2007	Afrotropical	Madagascan
*S.membranifera* Emery, 1869	Afrotropical	Australian, Madagascan, Nearctic, Neotropical, Oceanian, Oriental, Palearctic, Panamanian, Saharo-Arabian, Sino-Japanese
*S.minutula* Terayama & Kubota, 1989	Oriental, Sino-Japanese	Palearctic (not established)
*S.nepalensis* Baroni Urbani & De Andrade, 1994	Oriental	Madagascan (Mauritius), Sino-Japanese (Hong Kong, Macau)
*S.nigrescens* Wheeler, 1911	Panamanian	Panamanian (Cocos Island)
*S.perplexa* (Smith, 1876)	Australian	Australian (New Zealand)
*S.rogeri* Emery, 1890	Afrotropical	Madagascan, Nearctic, Neotropical, Oceanian, Oriental, Palearctic (not established), Panamanian
*S.silvestrii* Emery, 1906	Neotropical	Nearctic, Oriental?, Palearctic, Panamanian
*S.simoni* Emery, 1895	Afrotropical	Madagascan
*S.solifontis* Brown, 1949	Oriental, Sino-Japanese	Nearctic (not established)
*S.xenos* Brown, 1955	Australian	Australian (Lord Howe Island, New Zealand)

## Supplementary Material

XML Treatment for Strumigenys canina

XML Treatment for Strumigenys elegantula

XML Treatment for Strumigenys emmae

XML Treatment for Strumigenys exilirhina

XML Treatment for Strumigenys feae

XML Treatment for Strumigenys formosa

XML Treatment for Strumigenys heteropha

XML Treatment for Strumigenys hexamera

XML Treatment for Strumigenys hirsuta

XML Treatment for Strumigenys kichijo

XML Treatment for Strumigenys lantaui

XML Treatment for Strumigenys mazu

XML Treatment for Strumigenys membranifera

XML Treatment for Strumigenys minutula

XML Treatment for Strumigenys mitis

XML Treatment for Strumigenys cf. mutica

XML Treatment for Strumigenys nanzanensis

XML Treatment for Strumigenys nathistorisoc

XML Treatment for Strumigenys nepalensis

XML Treatment for Strumigenys rallarhina

XML Treatment for Strumigenys rogeri

XML Treatment for Strumigenys sauteri

XML Treatment for Strumigenys sydorata

XML Treatment for Strumigenys tisiphone
